# Hidden genetic variation shapes the structure of functional elements in *Drosophila*

**DOI:** 10.1038/s41588-017-0010-y

**Published:** 2017-12-18

**Authors:** Mahul Chakraborty, Nicholas W. VanKuren, Roy Zhao, Xinwen Zhang, Shannon Kalsow, J. J. Emerson

**Affiliations:** 1 Department of Ecology and Evolutionary Biology, University of California, Irvine, CA USA; 2 Department of Ecology and Evolution, University of Chicago, Chicago, IL USA; 3 Graduate Program in Mathematical, Computational and Systems Biology, University of California, Irvine, CA USA; 4 Center for Complex Biological Systems, University of California, Irvine, CA USA

**Keywords:** Genetics, Genomics

## Abstract

Mutations that add, subtract, rearrange, or otherwise refashion genome structure often affect phenotypes, although the fragmented nature of most contemporary assemblies obscures them. To discover such mutations, we assembled the first new reference-quality genome of *Drosophila melanogaster* since its initial sequencing. By comparing this new genome to the existing *D. melanogaster* assembly, we created a structural variant map of unprecedented resolution and identified extensive genetic variation that has remained hidden until now. Many of these variants constitute candidates underlying phenotypic variation, including tandem duplications and a transposable element insertion that amplifies the expression of detoxification-related genes associated with nicotine resistance. The abundance of important genetic variation that still evades discovery highlights how crucial high-quality reference genomes are to deciphering phenotypes.

## Abstract (web summary)

A reference-quality genome assembly of a *Drosophila melanogaster* strain allows for accurate mapping of structural variants through comparative analysis with the existing *Drosophila* genome. Previously hidden structural variation alters a larger fraction of the genome than SNPs and often affects candidate genes underlying complex traits.

## Main

Mutations underlying phenotypic variation remain elusive in trait-mapping studies^1^ despite the exponential accumulation of genomic data, suggesting that many causal variants are invisible to current genotyping approaches^2–5^. In fact, mutations like duplications, deletions, and transpositions^6,7^ are systematically under-represented by standard methods^7^, even as a consensus emerges that such structural variants (SVs) are important factors in the genetics of complex traits^2^. Addressing this problem requires compiling an accurate and complete catalog of the genomic features that are relevant to phenotypic variation, a goal most readily achieved by comparing nearly complete high-quality genomes^7^. Although the development of high-throughput short-read sequencing led to a steep drop in cost and a commensurate increase in the pace of sequencing^8^, it also led to a focus on single-nucleotide changes and small indels^3,9^. Paradoxically, this has also resulted in deterioration of the contiguity and completeness of new genome assemblies, due primarily to read-length limitations^10^.

**Fig. 1:**
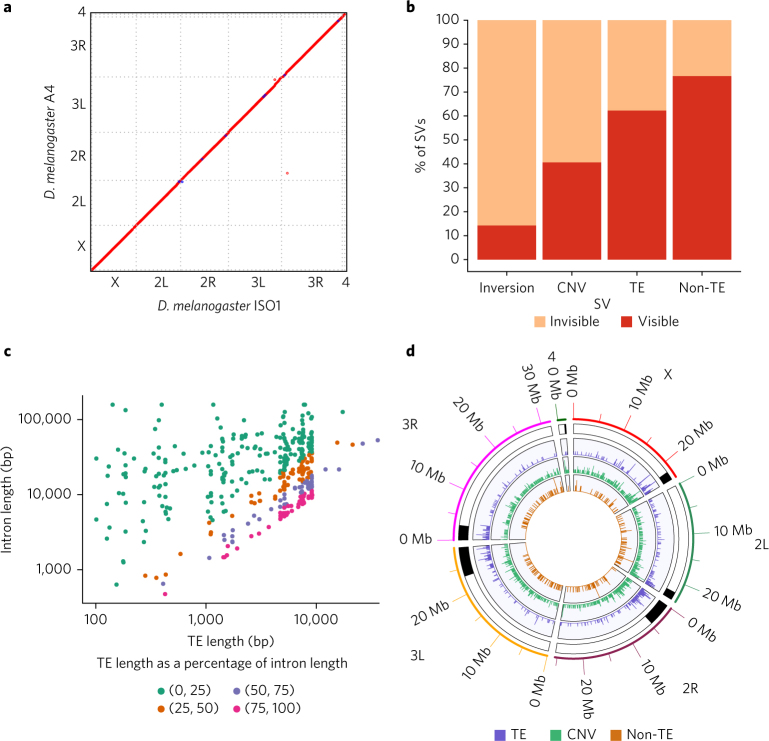
A4 assembly quality and structural variation. **a**, Dot plot between the *D. melanogaster* reference (ISO1) and A4 assemblies. The A4 assembly is as contiguous as the ISO1 assembly (scaffold N50 = 25.4 Mb versus 25.2 Mb; Supplementary Table 1). Repeats and TEs were masked to highlight the correspondence of the two genomes. **b**, The proportions of large (>100-bp) SVs in the A4 chromosome 2L assembly relative to the ISO1 2L assembly that were identified (visible) or missed (invisible) by short-read methods (Methods). **c**, Relationship between the lengths of TEs in ISO1 (median 5.1 kb) and the lengths of the introns into which they are inserted. Nearly equal intron and TE lengths indicate that many introns comprise mainly TEs. **d**, Distribution of SVs (>100 bp) across chromosome arms in the A4 genome. Track 1 shows pericentric heterochromatin (black). Tracks 2–4 show TEs, duplicate CNVs (relative to ISO1), and non-TE indels >100 bp in length, respectively. CNVs and TEs are present in higher densities in heterochromatin as compared to euchromatin, whereas non-TE indels are less numerous in heterochromatin.

Here we present a reference-quality assembly of a second *D. melanogaster* strain called A4 and introduce a comprehensive map of SVs, which identifies a large amount of hidden variation exceeding that due to SNPs and small indels, and which includes strong candidates to explain complex traits. The A4 strain is a part of the *Drosophila* Synthetic Population Resource (DSPR)^11^, a resource for mapping phenotypically relevant variants. We assembled the new A4 genome using high-coverage (147×) long reads through single-molecule real-time sequencing of DNA extracted from females (Supplementary Fig. 1), following an approach that has been shown to yield complete and contiguous assemblies^12^. The A4 assembly is more contiguous than release 6 of the ISO1 strain^13^—which is arguably the best metazoan whole-genome sequence assembly—with 50% of the genome contained in contiguous sequences (contigs) 22.3 Mb in length or longer (Supplementary Figs. 2 and 3). As compared to the ISO1 assembly, the A4 assembly comprises far fewer sequences (161 scaffolds versus 1,857 non-Y-chromosome scaffolds^14^) while maintaining comparable completeness (Supplementary Table 1)^15^. The two genomes are collinear across all major chromosome arms, making large-scale misassembly unlikely (Fig. 1a). An optical map of the A4 genome also supported the accuracy of the assembly (Supplementary Figs. 4 and 5).

We identified putative SVs by classifying regions of disagreement in a genome-wide pairwise alignment of the A4 and ISO1 assemblies as indels, copy number variants (CNVs), or inversions (Table 1). Reads spanning SVs showed that genotyping error was rare (<2.5%; Supplementary Table 2). However, because extremely long repeats are common in heterochromatin and require specialized approaches for assembly and validation^16^, we focused on euchromatin (Supplementary Table 3). We discovered 1,890 large (>100-bp) indels (Supplementary Fig. 6 and Supplementary Table 4), which affected more than 7 Mb. In contrast, mutations <100 bp in length affected only 1.4 Mb (indels, 722 kb; SNPs, 687 kb). Among large indels, 79% (1,486/1,890) were transposable element (TE) insertions (Supplementary Figs. 7–17). A previously published catalog of TE insertions in A4 based on 70× short-read coverage^17^ failed to find 38% of the TE insertions in A4 reported here (Fig. 1b, Supplementary Fig. 18, and Supplementary Table 5). These insertions, which are invisible to short-read approaches, often occur (in 34% of instances) when a TE is inserted near another TE, resulting in complex, non-uniquely mapping reads that are difficult to interpret. One such insertion was found in the A4 allele of the *MRP* gene (encoding multidrug-resistance-like protein 1), which is a candidate gene for resistance to the chemotherapy drug carboplatin^18^ (Supplementary Fig. 17).

**Table 1:** Number of different types of structural variants uncovered by aligning the A4 and ISO1 genomes

Mutation type (>100 bp)	Number of mutations in A4 euchromatin
Insertion (TE)	768
Deletion (TE)	718
Insertion (non-TE)	223
Deletion (non-TE)	181
CNV (more copies in A4)	209
CNV (fewer copies in A4)	181
Inversion	27

TE, transposable element; CNV, copy number variation.

We found that many TE insertions affected introns (395/718 in ISO1, 435/768 in A4), often greatly lengthening them (Fig. 1c and Supplementary Fig. 19). Additionally, TEs inserted into exons can be spliced out, effectively becoming new introns. We saw evidence of this in cDNA from ISO1^19^ and in RNA-seq reads in A4 that showed exon junctions flankng TE insertions (Supplementary Figs. 20–22 and Supplementary Table 6), which represents a genome-wide view of TE-derived introns segregating in a population. TE insertions within introns are associated with decreased transcription^20^, possibly caused by a phenomenon called intron delay, which slows transcription in long introns^21^. TE insertions can affect phenotype directly^22^, perhaps by modulating or disrupting the expression of important genes. Because most TEs are rare in *D. melanogaster* ^23^, they are poorly tagged by common variants, complicating genome-wide association study (GWAS) approaches for mapping traits; this mirrors similar complications in human GWAS^24^.

**Fig. 2:**
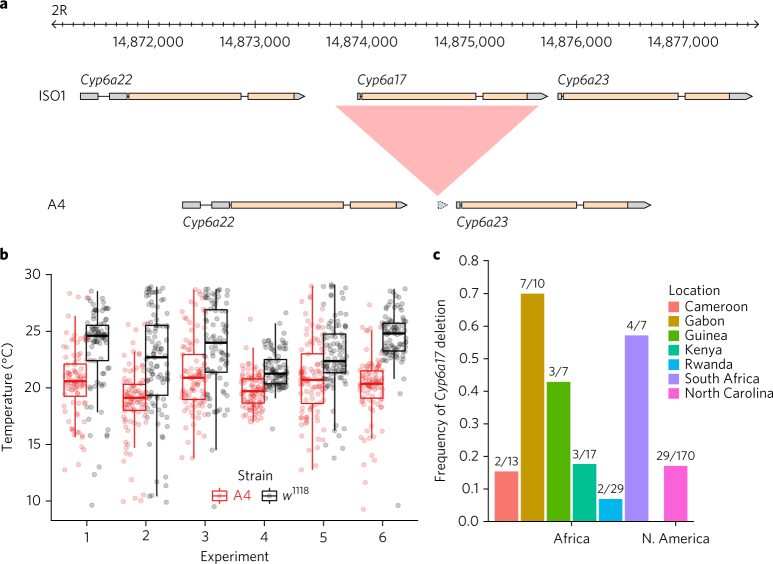
Copy number variation of *Cyp6a17* and its functional consequences. **a**, *Cyp6a17* is deleted in the A4 genome relative to the ISO1 genome. Alignment between annotated ISO1 and A4 assemblies on chromosome arm 2R shows a large ISO1 region (red) missing in A4. Gene models are shown (gray indicates noncoding sequences, and yellow indicates coding sequences). **b**, Temperature preference of strains A4 (∆*Cyp6a17*) and *w* ^1118^ (intact *Cyp6a17* ^23^). Preference was measured by recording the position of 100 flies along a linear 8 °C–30 °C temperature gradient after an adjustment period (Methods). Each dot represents the position of a fly along the gradient. Each experiment number is an independent pairwise trial. A4 flies occupy colder regions of the gradient than *w* ^1118^ flies (Fisher’s method on Wilcoxson rank-sum tests, meta *P* value << 10^−16^). Upper and lower hinges of the box plots represent 25% and 75% quantiles, respectively; the upper whisker indicates the largest observation less than or equal to the upper hinge + 1.5 times the interquartile range (IQR); the lower whisker indicates the smallest observation greater than or equal to the lower hinge – 1.5 times the IQR; and the middle horizontal bar indicates the median, 50% quantile. **c**, Frequency of the *Cyp6a17* deletion in African (DPGP2) and North American (DGRP) populations.

Non-TE insertions represented 20% of ISO1 and 23% of A4 insertions, and they accounted for 170 kb of sequence variation (Fig. 1d and Table 1). Although these mutations were much smaller than TEs (median 213 bp versus 4.7 kb), they often affected genes, and 23% even escaped detection by short reads (Fig. 1b). For example, among both hidden and visible deletions, there were 18 genes that were present in ISO1 and partially or completely absent in A4 (Supplementary Table 7), including *Cyp6a17* (Fig. 2a and Supplementary Fig. 23). Knockout of *Cyp6a17* in a previous study increased cold preference^25^. Indeed, A4 flies preferred colder temperatures than flies from a strain carrying an intact copy of *Cyp6a17* (Fig. 2b and Supplementary Fig. 24). Furthermore, this mutation was more common than expected for a deleterious allele (Fig. 2c), suggesting that it has a role in regulating how flies respond to temperature in the wild. One deletion missed by short-read genotyping removed the second exon of *Mur18B* (and 41 amino acids of the encoded chitin-binding protein that confers resistance to high-temperature stress^26^) (Supplementary Fig. 25), likely rendering the A4 *Mur18B* allele defective.

We discovered 27 inversions, ranging from 100 bp to 21 kb in length (Supplementary Table 4), that affected 60 kb of sequence, only 4 of which were detected by paired-end methods (Fig. 1b and Supplementary Table 5). These inversions often (in 21/27 instances) affected regions harboring genes, including a 21-kb region that spanned five genes encoding gustatory receptors: *Gr22a*, *Gr22b*, *Gr22c*, *Gr22d*, and *Gr22e* (Supplementary Table 4). Although such clusters of related sequences may obscure the read-mapping information used to detect inversions, we could not find genomic features that might explain why the other inversions were missed. The A4 optical map identified a putative inversion occupying 300 kb of the proximal end of the X-chromosome scaffold that was not resolved by the A4 assembly (Supplementary Figs. 4 and 5). Failure to resolve this inversion is not unexpected because assembly methods tuned for euchromatin perform poorly in heterochromatic regions^16^.

**Fig. 3:**
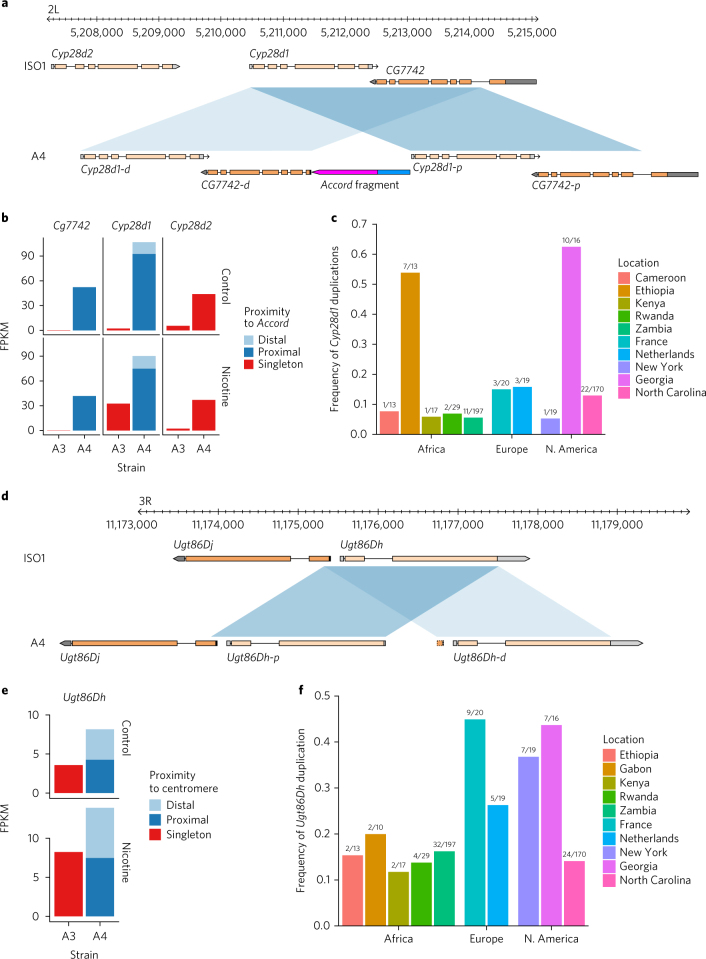
Copy number variation in *Ugt86Dh* and *Cyp28d1* and its effect on gene expression variation. Shaded parallelograms (light blue, distal copy; dark blue, proximal copy) correspond to the single and duplicated regions in ISO1 and A4. **a**, Schematic showing duplication of *Cyp28d1* and *CG7742* in A4. ISO1 and strain A3 possess one copy of *Cyp28d1*, whereas A4 has two copies. A 1.5-kb *Accord* fragment (pink) containing an LTR (blue) is located between the proximal *Cyp28d1* and the distal *CG7742*. Gene models are shown with gray (noncoding) and orange (coding) rectangles. **b**, Paralog-specific expression of candidate QTL genes at Q1 in A4 and A3 in the presence and absence (control) of nicotine in the food. *CG7742* and *Cyp28d1* copies located nearer the *Accord* element are transcribed at higher levels than those that are more distal. FPKM, fragments per kilobase of transcript per million mapped reads. **c**, Combined frequency of four *Cyp28d* duplicate alleles in African (DPGP2 and DPGP3) and North American populations. **d**, Schematic showing that tandem duplication of *Ugt86Dh* in A4 created *Ugt86Dh-d*. **e**, Paralog-specific expression of candidate QTL gene Ugt86Dh in A4 and A3 in the presence and absence (control) of nicotine in the food. In contrast to *Cyp28d1* duplicates, the two copies of *Ugt86Dh* are expressed at similar levels, and their expression nearly doubles in the presence of nicotine. **f**, Frequency of the *Ugt86Dh* duplicate in African (DPGP2 and DPGP3) and North American populations.

We discovered 390 CNVs (209 in A4 and 181 in ISO1) that affected ~600 kb (Fig. 1d, Supplementary Figs. 26–36, and Supplementary Table 4). Although some CNVs were missed by paired-end methods owing to spacer sequences between copies that were longer than the library fragments (Fig. 3a,d), most (~90%) of the CNVs were missed because they occurred in complex tandem repeats (Supplementary Fig. 37). Unlike indels, most CNVs (64%) affected exons. Additionally, short-read CNV genotyping methods missed 13 of 34 protein-coding genes that were duplicated in A4. In total, only ~40% of CNVs were discoverable with high-specificity split-read and read-orientation methods^27,28^ (Fig. 1b and Supplementary Fig. 38). Consistent with previous observations^29^, coverage-based methods were extremely nonspecific (Supplementary Fig. 38) and were therefore excluded from analysis. We next compared published gene expression data from larvae of A4 to expression data for a DSPR strain called A3^30^ and identified 17 A4 duplicate genes that are single copy in ISO1 with increased expression (Supplementary Table 8), including genes previously identified as candidates for cold adaptation, olfactory response, and toxin resistance, among others (Fig. 3a,d and Supplementary Tables 8 and 9). Notably, eight of these CNVs were invisible to short-read methods (Supplementary Table 8).

A longstanding concern in trait-mapping studies is failure to genotype candidate mutations^2^. Because A4 is a parental line of the DSPR trait-mapping panel^11^, we could confront this problem directly. Among the eight duplicate genes with increased expression in A4 that escaped detection, *Cyp28d1* and *Ugt86Dh* fell under quantitative trait loci (QTLs) for resistance to nicotine, a plant defense toxin^30,31^. One QTL (Q1) contains two genes, *Cyp28d1* and *Cyp28d2*, that encode cytochrome P450 enzymes, both of which were upregulated^30^. The other candidate region that showed a major effect contains the *Ugt86D* gene cluster, which includes several differentially regulated genes, including *Ugt86Dh* (Fig. 3d,e). Candidate mutations like these are of obvious interest to researchers trying to dissect any trait, and yet they were not visible in the initial study^30^.

In the A4 assembly, Q1 contains a 3,755-bp tandem duplication in which the duplicated regions are separated by a 1.5-kb spacer, resulting in two copies of *Cyp28d1* (Fig. 3a and Supplementary Figs. 39–41). We compared paralog-specific expression levels of the *Cyp28d1* copies in A4 to expression of the single copy in A3. In the absence of nicotine, the proximal and distal copies in A4 exhibited ~41-fold and ~6.3-fold higher expression, respectively, than the single copy in A3 (Fig. 3b). The intervening spacer sequence proved to be the 5′ end of *Accord*, a long terminal repeat (LTR) retrotransposon (Fig. 3a). Insertion of *Accord* upstream of another gene called *Cyp6g1* has been linked to upregulation of the encoded cytochrome P450 enzyme^32^, suggesting that the retrotransposon may be responsible for the upregulated expression rather than the tandem duplication of the *Cyp28d* gene. The second nicotine-resistance QTL contains several *Ugt* genes, including *Ugt86Dh*, which have previously been implicated in increased resistance to the pesticide DDT^33^. Of note, we found that *Ugt86Dh* was duplicated in A4 (Fig. 3d and Supplementary Figs. 42 and 43); this mutation escaped detection by paired-end short reads (Supplementary Table 5). Although several *Ugt* genes in the Q4 QTL showed higher expression in nicotine-resistant A4 larvae than in sensitive A3 larvae^30^ (Fig. 3e), candidate variants that explain these differences have yet to be identified.

Because nicotine analogs are widely used pesticides, we predict that resistance-conferring mutations are common, mirroring observations for DDT. Indeed, we found that four duplicate alleles spanning *Cyp28d1* and *Cyp28d2* segregated at intermediate to high frequencies in multiple populations (Fig. 3c) in a 25-kb region where we expected duplicate heterozygosity to be less than 0.1. Similarly, the single duplicate allele of *Ugt86Dh* segregated at high or intermediate frequency in nearly all of the populations we examined^6^ (Fig. 3f). Finally, patterns of SNP variation surrounding both *Cyp28d1* and *Ugt86Dh* are consistent with recent bouts of natural selection (Supplementary Figs. 44 and 45), suggesting recent adaptation to nicotinoids.

Although we focus on genetic variation in A4 relative to ISO1, there is no biologically meaningful sense in which any individual of a species is a more appropriate reference than another. Yet, despite the prevalence of heritable phenotypic variation, functional work often describes results derived from individuals with diverse genotypes as applying to an entire species^34^. Approaches like RNA interference (RNAi) or gene editing with CRISPR require precise sequence information about their targets and can be easily misled by hidden structural variation. One study on the origin of new genes in *D. melanogaster* argues that new genes rapidly become essential, and the authors even report a new gene called *p24-2* that is so young that it is present in only *D. melanogaster* ^35^. Experiments targeting *p24-2* using RNAi constructs suggested that, although new, *p24-2* is essential. However, *p24-2* was absent in eight of the ten strains we examined, including A4 and Oregon-R (Supplementary Figs. 46 and 47), which calls into question its essential nature in *D. melanogaster*. Because the original construct actually targeted both *p24-2* and its essential paralog *eca* ^36,37^ (Supplementary Note), we tested two other constructs targeting *p24-2*, neither of which resulted in any reduction in viability (Supplementary Table 10), thus bolstering the suggestion that *p24-2* is not essential.

The ubiquity of hidden variation in genome structure is merely an indication of the extent of the underlying genetic variation governing phenotypes. Together with careful phenotypic measurements, a new generation of high-quality genomes will identify previously invisible heritable phenotypic variation. Our results show that popular genotyping approaches miss a significant fraction of SVs (Fig. 1b, Supplementary Figs. 18 and 38, and Supplementary Table 5), including ones that affect gene expression and organismal phenotype (Supplementary Tables 8 and 9), suggesting that previous estimates of the contribution of SVs to regulatory^38^ and phenotypic variation are misleading^39^. The extensive hidden variation we observe segregates in *D. melanogaster*, a species that likely harbors fewer complex structural features than humans or livestock, as well as crop species like wheat and maize. Consequently, we suggest that the true medical and agricultural impact of structural variation is likely to be much greater than the already considerable estimates made without recourse to multiple reference-grade assemblies^29^.

## Methods

### DNA sequencing and genome assembly

A4 DNA was extracted from females and used in SMRTbell library preparation as described previously^12^. We sequenced this library on 30 SMRTcells using P6-C4 chemistry on a Pacific Biosciences RSII platform at the University of California High-Throughput Genomics Facility, yielding 18.7 Gb of sequence. We then followed the method described previously^12^ to assemble the A4 genome. We assembled a draft genome using PBcR-MHAP^40^ in wgs 8.3rc1 and PacBio reads (NG50 = 13.9 Mb, 147 Mb in total; NG50 is the contig length such that 50% of an assumed assembly size is contained within contigs of this length or longer) and then generated a hybrid assembly with DBG2OLC^41^ using the longest 30× PacBio reads and 75× paired-end Illumina reads from ref. ^42^ (assuming a genome size of 130 Mb; NG50 = 4.23 Mb, 129 Mb in total). We merged the two assemblies using quickmerge v0.1 with default settings, except hco = 5, c = 1.5, and l = 2 Mb. The merge yielded an assembly (NG50 = 21.3 Mb, 130 Mb in total) that was both smaller than expected^42^ and smaller than the PacBio-only assembly. Therefore, we added contigs that were unique to the PacBio assembly to the hybrid assembly using quickmerge as described above but with I = 5 Mb. Finally, we generated the final assembly by running finisherSC^43^ with default settings, polishing the assembly twice with quiver (SMRT Analysis v2.3), and with Pilon v1.3^44^ (using A4 reads from ref. ^42^). This yielded a final assembly of 144 Mb with N50 = 22.3 Mb (Supplementary Table 1).

### Bionano data

A4 embryos less than 12 h old were collected on Petri dishes containing apple juice and agar, dechorionated using 50% bleach, rinsed with water, and stored at –80 °C. DNA was extracted from frozen embryos using the Animal Tissue DNA Isolation kit (Bionano Genomics). Bionano Irys optical data were generated and assembled with IrysSolve 2.1 at Bionano Genomics. We then merged the Bionano assembly with the final assembly contigs (described in “DNA sequencing and genome assembly”) using IrysSolve, retaining Bionano assembly features when the two assemblies disagreed.

### Comparative scaffolding

The scaffold for the A4 assembly was prepared with the software mscaffolder (see URLs) using the release 6 *D. melanogaster* genome (r6.09) assembly^13^ as the reference. Prior to scaffolding, TEs and repeats in both assemblies were masked using default settings for RepeatMasker (v4.0.6). The repeat-masked A4 assembly was aligned to the repeat-masked major chromosome arms (X, 2L, 2R, 3L, 3R, and 4) of the *D. melanogaster* ISO1 assembly using MUMmer^45^. Alignments were further filtered using the delta-filter utility with the -m option, and the contigs were assigned to specific chromosome arms on the basis of the mutually best alignment. Contigs showing less than 40% of the total alignment for any chromosome arms could not be assigned a chromosomal location and therefore were not scaffolded. The mapped contigs were ordered on the basis of the starting coordinate of their alignment that did not overlap with the preceding reference chromosome–contig alignment. Finally, the mapped contigs were joined with 100 Ns, a convention representing assembly gaps. The unscaffolded sequences were named with a ‘U’ prefix.

### Benchmarking universal single-copy orthologs (BUSCO) analysis

We used BUSCO (v1.22)^15^ to evaluate the completeness and accuracy of the A4 and ISO1 release 6 assemblies. ISO1 contains five BUSCOs (BUSCOaEOG75R3J9, BUSCOaEOG7SJRJ9, BUSCOaEOG7SJRK2, BUSCOaEOG7WMR0H, and BUSCOaEOG71S8ZH) that are missing from the A4 assembly. To validate the absence of these five BUSCOs in the A4 assembly, the full-length sequences of the ISO1 genes (*Ftz-f1*, *CG7627*, *Raw*, *Maf1*, and *Cv-*c) were downloaded from FlyBase^14^ and queried against the A4 assembly with MUMmer. MUMmer found all five ‘missing’ BUSCOs in the A4 assembly in single copies. The BUSCO counts for A4 were adjusted accordingly.

### Structural variant detection

*Detection of CNVs via whole-genome alignment*. We aligned the ISO1 and A4 assemblies using MUMmer^45^ (mummer -mumreference -l 20 -b) and then clustered maximal exact matches (MEMs) between the two mgaps (mgaps -C -s 200 -f 0.12 -l 100). The l parameter in mgaps was set to 100 to detect duplicates that were 100 bp or longer. We used a pipeline called svmu (structural variants from MUMmer; see URLs) to automate CNV detection from overlapping mgaps clusters. When reference sequence regions in two separate alignment clusters overlapped, the overlapping segment of the reference sequence regions was inferred to be duplicated in the query sequence. This approach can also identify (i) a duplicated sequence that is present in both the genomes but has diverged owing to the presence of repeats or indels and (ii) CNVs containing TE sequences. We filtered the latter using RepeatMasker (v4.0.6). We identified false-positive duplication calls by aligning the putatively duplicated reference sequences back to the ISO1 and A4 genomes using nucmer (nucmer --maxmatch --g 200) and then counting the copy number using checkCNV, which is also included in the svmu pipeline. svmu was run with the default parameters; checkCNV was run with c = 500 (max copy number 500), qco = 10,000 (10 kb of insertion or deletion allowed within a copy), and rco = 0.2 (unaligned length of up to 20% of the sequence length between reference and query copies allowed). CNVs occurring within 2 kb of each other were designated as ‘complex events’ and combined (bedtools merge --d 2000)^46^ for the purpose of counting the total number of CNVs present in the genome (Supplementary Table 11). However, the total sequence affected by CNVs was counted before merging. Functional annotation of CNVs was based on gene annotation of ISO1 release 6.

### Detection of indels via whole-genome alignment

Insertions (>100 bp) in the A4 genome appear as alignment gaps between two adjacent syntenic blocks when ISO1 is aligned to A4 (and vice versa). We aligned the A4 sequence to the ISO1 sequence using nucmer (default parameters) and then identified adjacent syntenic blocks with gaps >100 bp in length between them in the A4 assembly but <10% the gap length in the ISO1 assembly. Indel detection was carried out with the svmu utility findInDel. A deletion was inferred for a specific gene (e.g., *Cyp6a17*) when an ortholog of the gene was present in the closely related species *Drosophila simulans*.

### Detection of inversions via whole-genome alignment

We identified inversions in the A4 genome by aligning it to the ISO1 genome using nucmer (-mumreference) and then processing the outputted delta file using findInDel. A4 regions that ran in the reverse direction with respect to the ISO1 sequence were recorded as inversions. TEs were removed from this list using RepeatMasker annotations for ISO1.

### Genotyping CNVs, indels, and inversions using Illumina reads

Three common, complementary strategies are typically used to discover CNVs with paired-end Illumina reads: read depth, read-pair mapping orientation, and split-read mapping^7^. We identified duplications (100 bp to 25 kb long) in the A4 genome using 70× paired-end reads^11^ with CNVnator^47^ for the read depth approach, pecnv^28^ for the read-pair orientation approach, and Pindel^27^ for the split-read mapping approach. We mapped reads to ISO1 release 6 using bwa-mem for CNVnator and pindel and bwa-aln for pecnv^48^. We required at least three supporting read pairs for pecnv calls^28^ and used a bin size of 100 for CNVnator because of the data’s high coverage. Furthermore, we used CNVnator and Pindel to identify large (>100-bp) indels and Pindel to identify inversions. We manually compared these short-read-based calls to our alignment-based CNV calls for all of chromosome arm 2L.

TE insertion coordinates for A4 were obtained from DSPR (http://wfitch.bio.uci.edu/~dspr/). We manually compared our TE insertion calls and those from ref. ^17^ for all of chromosome arm 2L.

### SNP and small indel detection

SNPs and small (<100-bp) indels in the A4 assembly were identified using the show-snps utility from MUMmer^45^. We aligned A4 scaffolds to ISO1 scaffolds using nucmer (-mumreference) and then filtered repeats using delta-filter in conjunction with the --r and --q options. SNPs and small indels were called from the filtered data using show-snps with --Clr options.

### Validation of duplicates and indels

Dot plots between A4 and ISO1 for all SV loci on chromosome arm 2L were manually inspected to confirm the accuracy of the MUMmer-based genotyping. All manually inspected loci corresponded to the automated genotype calls. To quantify the effect of assembly errors in A4 on SV calls, we required that unassembled, corrected long reads from A4 agree with the A4 assembly in the region spanning the entire mutation. To do this, we mapped the PBcR-MHAP-corrected long reads to the A4 assembly using blasr v1.3.1.142244 (-bestn 1 --sam) and identified all of the reads that spanned the mutation-containing region with anchors in the flanking sequence of at least 250 bp on each side. For our stringent validation criteria, we required at least two fully spanning reads to overlap each SV (Supplementary Fig. 48a). These fully spanning reads were required to have at least 99.5% alignment coverage (*P*_aligned_) and less than a ratio of 0.005 of gaps to read length (*R*_gaps_; Supplementary Fig. 48a). For our standard validation criteria, we permitted validation under the following relaxed criteria: (i) overlap-spanning reads (at least two on each side) that otherwise fit the stringent criteria above and (ii) fully spanning reads with at least 97.5% alignment coverage (*P*_aligned_) and less than a ratio of 0.025 of gaps to read length (*R*_gaps_; Supplementary Fig. 48b).

Half of our sequencing data were present in reads that were 17,885 bp or longer, which was enough to achieve more than 60-fold coverage across the entirety of the euchromatin and more than 10-fold coverage of the genome in reads that were 30 kb or longer. Such long reads contained unique sequences flanking each side of the mutation, as well as the mutation breakpoints and the mutation itself, making this a powerful approach to validating SV calls.

### PCR validation

We assayed for the presence and absence of *Cyp28d1* and *p24-2* copies using PCR (Supplementary Figs. 41 and 47, and Supplementary Table 12). We extracted DNA from 25 flies from each strain using the Magattract HMW DNA kit (Qiagen), and we used Phusion (New England Biolabs) for PCRs that had an amplification time of 15 s for the *Cyp28d1* reactions and 30 s for the *p24-2* reactions.

### Temperature-preference assay

We created a linear temperature gradient on a solid aluminum bar (total dimensions: 24 inches × 4 inches × 4 inches) by placing 4 inches of one end of the bar inside a reservoir containing ice water (0 °C) and 4 inches of the other end inside a reservoir containing warm water (35 °C) (Supplementary Fig. 24). This left ~40 cm of aluminum bar exposed between the baths. Temperatures along the bar were measured by 11 temperature sensors (Tmp36 analog temperature sensors from Adafruit) that were evenly spaced at 4-cm intervals and sealed into holes drilled into the bar after being secured with thermal epoxy (OMEGABOND 101 Two-Part Epoxy). The probes were connected to three four-channel 16-bit analog-to-digital converters (ADS1115 from Adafruit), which were in turn calibrated and monitored by a Raspberry Pi 3 single-board computer. Automated temperatures were recorded every second using a custom Python script (see URLs) during the experiment to verify the stability of the gradient. The temperature measurements at the end of the experiment were used in assigning temperatures to individual flies. The temperature gradient on the aluminum bar ranged from 9 °C to 30 °C (Fig. 2b). We compared the preference of A4 flies, which lack the *Cyp6a17* gene, to that of *w* ^1118^ flies (BDSC stock 5905), which have an intact copy of *Cyp6a17* ^25^. We collected groups of 100 1- to 3-d-old flies of mixed sex and kept them at 25 °C for 24 h. Before the assay, flies were immobilized with light anesthesia and placed between a thin aluminum sheet cut into the shape of the aluminum bar surface and an acrylic lid possessing a partition to create two ‘lanes’ for the flies to behave without interacting with each other. Quinine sulfate was applied to the roof and walls of each channel in the lid so that the flies would avoid these surfaces and be constantly in contact with the aluminum surface. Flies were allowed to recover on the aluminum sheet in a 25 °C incubator for 40 min after being anesthetized. The aluminum sheet was then placed on top of the aluminum bar and left for 40 min in the dark. A photo was taken to record the positions of the flies on the block after 40 min. We recorded fly positions and interpolated their temperatures using linear regression based on temperature-probe readings.

### Statistical analyses

We replicated the temperature preference assay experiment six times. Three replicates were conducted with A4 flies in lane 1 and *w* ^1118^ flies in lane 2, and three replicates were conducted with the lane assignments reversed. We performed a nonparametric Wilcoxon rank-sum test, which does not assume a particular distribution for the data, on each of these six replicates to test for a difference in temperature preference between the two strains. These six individual tests produced *P* values of 2.12 × 10^–10^, 6.76 × 10^–10^, 1.89 × 10^–6^, 9.21 × 10^–14^, 1.96 × 10^–6^, and 1.25 × 10^–24^. To obtain a combined *P* value, we performed a meta-analysis using Fisher’s method, which gave a very low meta *P* value (*P* << 10^−16^).

### RNAi strain construction and screening

Strain 60100 (Vienna *Drosophila* Resource Center) contains two attP sites at 2L: 22,019,296 (near tiptop; VIE260B) and 2L: 9,437,482 (VIE260B-2). Activation of RNAi constructs inserted into VIE260B results in ectopic activation of tiptop and phenotypes independent of the RNAi target^49^. PCR screening showed that KK109179 contained insertions at both sites and likely caused the lethal phenotype observed in ref. ^35^ (Supplementary Fig. 49). We removed the insertion at VIE260B following the crossing scheme outlined by ref. ^49^ and kept two of the resulting lines with insertions only at VIE260B-2 (Supplementary Fig. 49).

We generated a new *p24-2* RNAi line as previously described^50^. We designed the RNAi construct CG33105_RNAi using the E-RNAi server (see URLs). CG33105_RNAi was the only possible construct >50 bp in length with 100% of the possible 19-mers uniquely matching *p24-2*. CG33105_RNAi was cloned into pKC26 and then injected into flies from strain 60100 at 250 ng/μl. We isolated transformants using Bloomington *Drosophila* Stock Center (BDSC) balancer stock 9325 to ensure that the RNAi construct was inserted only at VIE260B-2 using PCR54. NV-CG33105-2 and NV-CG33105-6 are derived from different transformants, but carry the same CG33105_RNAi construct. We drove RNAi expression using lines that constitutively expressed GAL4 under the control of the *Act5C* or *αTub84B* promoter (BDSC lines 4414 and 5138, respectively). Five males and five virgin driver females were allowed to cross for 9 d at 25 °C and a 12-h light/12-h dark cycle; they were then removed from the vials. F_1_ progeny flies were counted 19 d after crossing. The proportion of wild-type (RNAi-active) F_1_ flies was compared to the proportion of wild-type F_1_ flies from control crosses between 60,100 males and the driver strains. We confirmed presence of the *p24-2* duplicate in each of these lines using PCR (Supplementary Table 12) and Sanger sequencing.

### Expression analysis

Genome-wide gene expression differences between A3 and A4 larvae were analyzed as described previously^30^. Sequences of the genes from A3 larvae were obtained from an A3 genome assembly constructed with publicly available A3 Illumina paired-end reads. To compare the expression levels of *Cyp28d1*, *CG7742*, and *Ugt86Dh* gene copies, we aligned publicly available 100-bp RNA-seq reads^30^ to A4 mRNA sequences using Bowtie2^51^ (with --score-min L,0,0 to ensure that only perfectly aligned unique (i.e., copy-specific) reads were kept for FPKM calculations). We adjusted transcript length by subtracting the length of regions to which no SNP-covering read aligned because only reads overlapping the SNPs could be included in FPKM calculations. For example, *Cyp28d1* gene copies are distinguishable by 15 SNPs. When regions that cannot be spanned by perfectly aligned unique reads are removed from the effective transcript length, 310 bp is subtracted from the total 1,509-bp transcript length, leaving an effective transcript length of 1,199 bp. Similarly, for *Ugt86Dh* and *CG7742*, transcript lengths of 1,065 bp and 755 bp were used to calculate FPKM values, respectively. No such adjustments were made for the single-copy genes not segregating for duplications. The total number of reads aligned to the genomes was calculated based on alignment of the single-end RNA-seq reads aligned to the A4 and A3 genomes using TopHat^52^.

### Testing for selective sweeps

We used the composite likelihood ratio (CLR) statistic of SweepFinder2 v1.0 to test for recent selective sweeps^53,54^. CLR values were calculated using the frequency of SNPs present in each sample over a grid with 250-bp increments. Sites were polarized using *D. simulans*, *Drosophila yakuba*, and *Drosophila erecta*. Invariant sites that differed from the inferred ancestral state (substitutions) were included in the analysis, thus improving power and robustness to bottlenecks^53,55^. The significance of the results was evaluated by comparing the CLR values to 100 coalescent neutral simulations generated using ms^56^. Estimates of the effective population size, neutral mutation rate, and recombination rate were taken from previous publications^57^. The 95% confidence intervals were computed using the largest CLR values from each neutral simulation.

### Estimating frequencies of duplicate alleles

The frequency of duplicate alleles was estimated from next-generation Illumina data (Supplementary Note) by analyzing the density of divergently mapped read pairs. Reads were mapped against the release 6 ISO1 reference genome using bwa-mem^48^. Divergent read pairs were selected by taking the complement of paired reads in the BAM file that mapped with proper orientation, defined as pairs of reads that mapped to the same chromosome on opposite strands and were flagged by the aligner as being properly aligned with respect to each other. Duplications were called for samples that showed a clear peak and high signal-to-noise ratio in the coverage density for divergent read pairs at breakpoints surrounding genes that were found to be duplicated in the A4 sequence. The divergent read pair signals for several duplicate alleles for *Cyp28d1* from various populations are shown in Supplementary Fig. 50. Samples with low genomic coverage (<10 Mb over the chromosome containing the duplication) or inferred to be identical by descent to other samples over a region containing the duplication, using estimates of homozygous coverage and identity by descent from ref. ^58^, were excluded from analysis. Populations were excluded from this analysis if they contained fewer than ten samples.

### URLs

All codes used for variant calling and scaffolding have been deposited to GitHub (https://github.com/mahulchak). Codes used in the temperature-gradient experiment have been deposited to GitHub (https://github.com/jjemerson/TemperatureGradient). RNAi was designed using the E-RNAi server at http://www.dkfz.de/signaling/e-rnai3/.

### Life Sciences Reporting Summary

Further information on experimental design is available in the Life Sciences Reporting Summary.

### Data availability

All single-molecule sequence data have been deposited to the NCBI Sequence Read Archive (SRA) and can be found under accession SRX2729308. The A4 scaffolded assembly has been deposited in the NCBI Assembly database under accession GCA_002300595.1. All the variant calls are provided in the supplementary files.

## Methods

Methods, including statements of data availability and any associated accession codes and references, are available at 10.1038/s41588-017-0010-y.

## Supplementary information

Supplementary Text and Figures Supplementary Figures 1–50, Supplementary Tables 1, 3, 9, 10, and 12, and Supplementary Note

Life Sciences Reporting Summary

Supplementary Table 2 Validation summary of the genomic intervals containing SVs

Supplementary Table 4 Coordinates of the CNVs, indels, and inversions in A4 and ISO1

Supplementary Table 5 CNVs and indels for chromosome arms 2L as called by various CNV-calling software

Supplementary Table 6 Summary of putative TE introns

Supplementary Table 7 Genes mutated by non-TE indels in A4

Supplementary Table 8 Expression changes in genes in A4 with increased copy number

Supplementary Table 11 The unmerged CNV calls from A4–ISO1 genome alignment
