# Supplementary material for: Hidden genetic variation shapes the structure of functional elements in Drosophila
Source: Nat Genet. 2017 Dec 18;50(1):20–5. doi: 10.1038/s41588-017-0010-y (PMC5742068; doi:10.1038/s41588-017-0010-y)
Supplement: Supplementary file 1 — Supplementary Figures 1–50, Supplementary Tables 1, 3, 9, 10, and 12, and Supplementary Note [file 41588_2017_10_MOESM1_ESM.pdf]

nature genetics

SUPPLEMENTARY INFORMATION
https://doi.org/10.1038/s41588-017-0010-y

In the format provided by the authors and unedited.

Hidden genetic variation shapes the structure of functional elements in *Drosophila*

Mahul Chakraborty[1*], Nicholas W. VanKuren[2], Roy Zhao[3,4], Xinwen Zhang[1,3], Shannon Kalsow[1] and J. J. Emerson[1,4*]

[1]Department of Ecology and Evolutionary Biology, University of California, Irvine, CA, USA. [2]Department of Ecology and Evolution, University of Chicago, Chicago, IL, USA. [3]Graduate Program in Mathematical, Computational and Systems Biology, University of California, Irvine, CA, USA. [4]Center for Complex Biological Systems, University of California, Irvine, CA, USA. *e-mail: mchakrab@uci.edu; jje@uci.edu

© 2017 Nature America Inc., part of Springer Nature. All rights reserved.

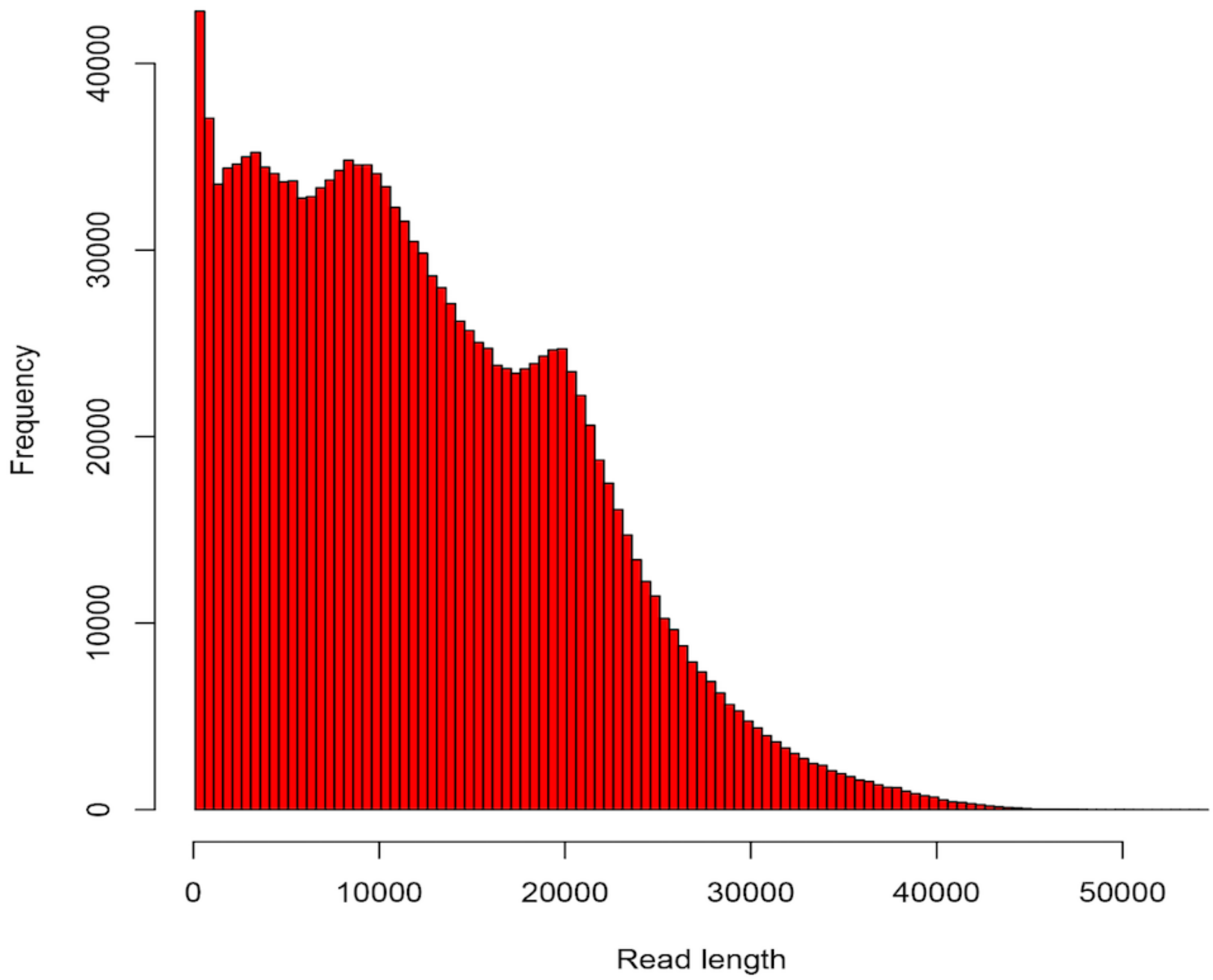

Supplementary Figure 1. Distribution of A4 PacBio long reads. Half of total coverage is contained within reads at least 18 kbp long (i.e. NR50 = 18 kbp).

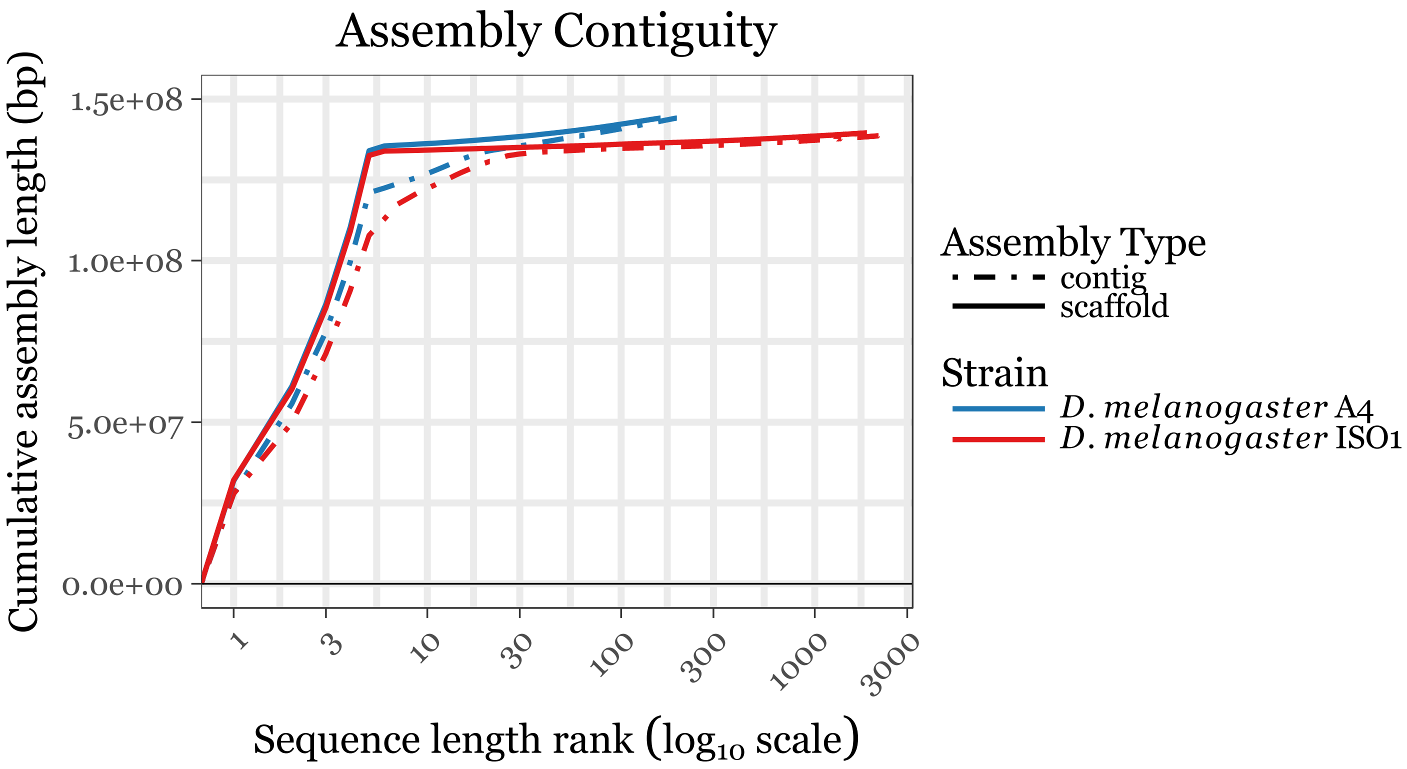

Supplementary Figure 2. Cumulative sequence length distributions for the final A4 and FlyBase release 6 ISO1 assemblies. The X-axis is the sequence length rank sorted in descending order. The Y-axis is the cumulative length of all sequences to the rank on the X-axis. A4 is more contiguous than ISO1 on both the contig and scaffold levels. The total amount of genome assembled for A4 is also about 4 Mbp more than for ISO1, as indicated by the A4 curves reaching a higher Y-value. The mitochondrial genome was excluded from both genomes and Y-chromosome sequences were excluded from the ISO1 genome (the A4 genome was derived from females and therefore has no Y-chromosome sequence).

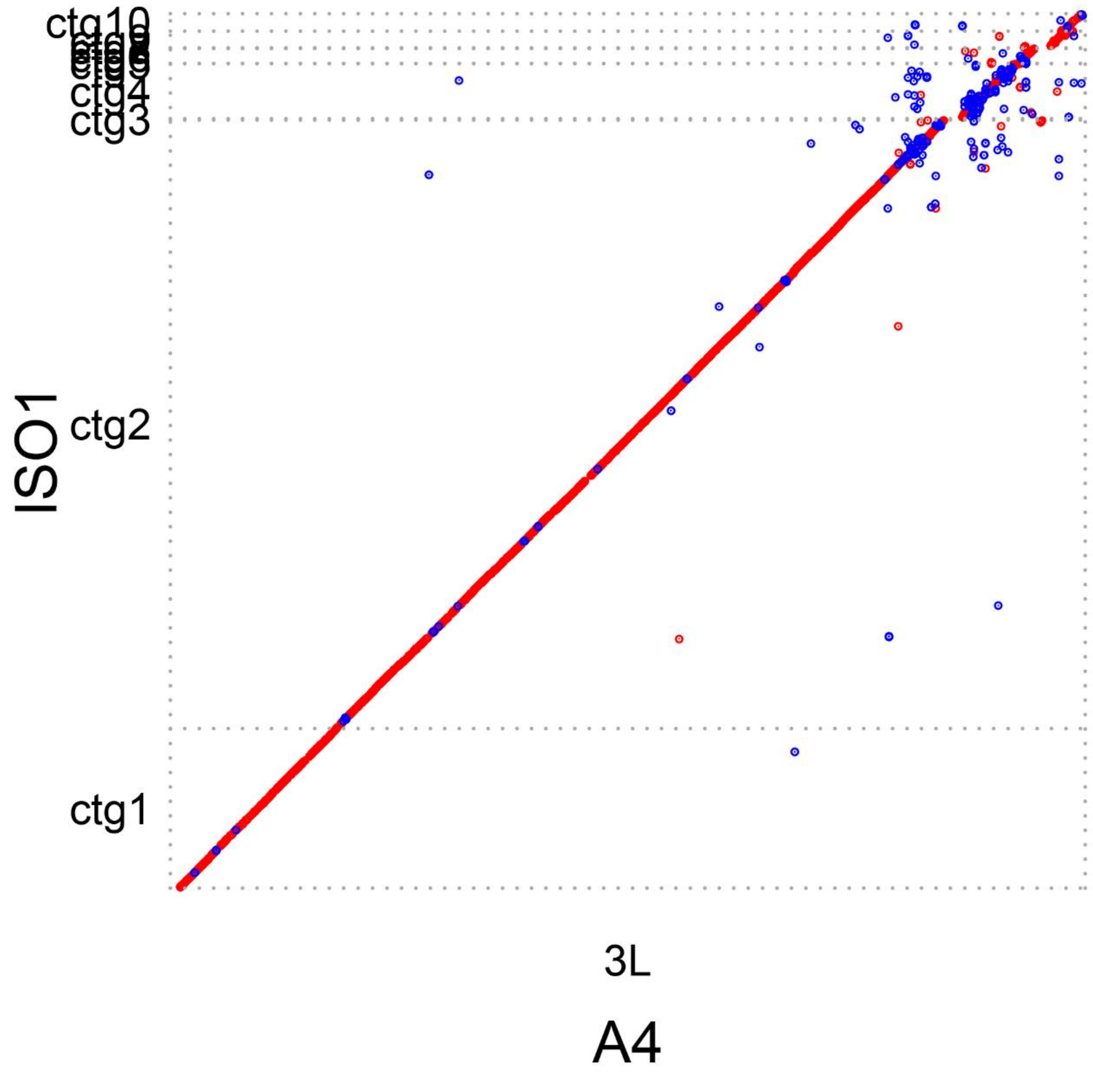

Supplementary Figure 3. Alignment dot plot between the 3L contigs in ISO1 and a single 28Mb contig in A4.

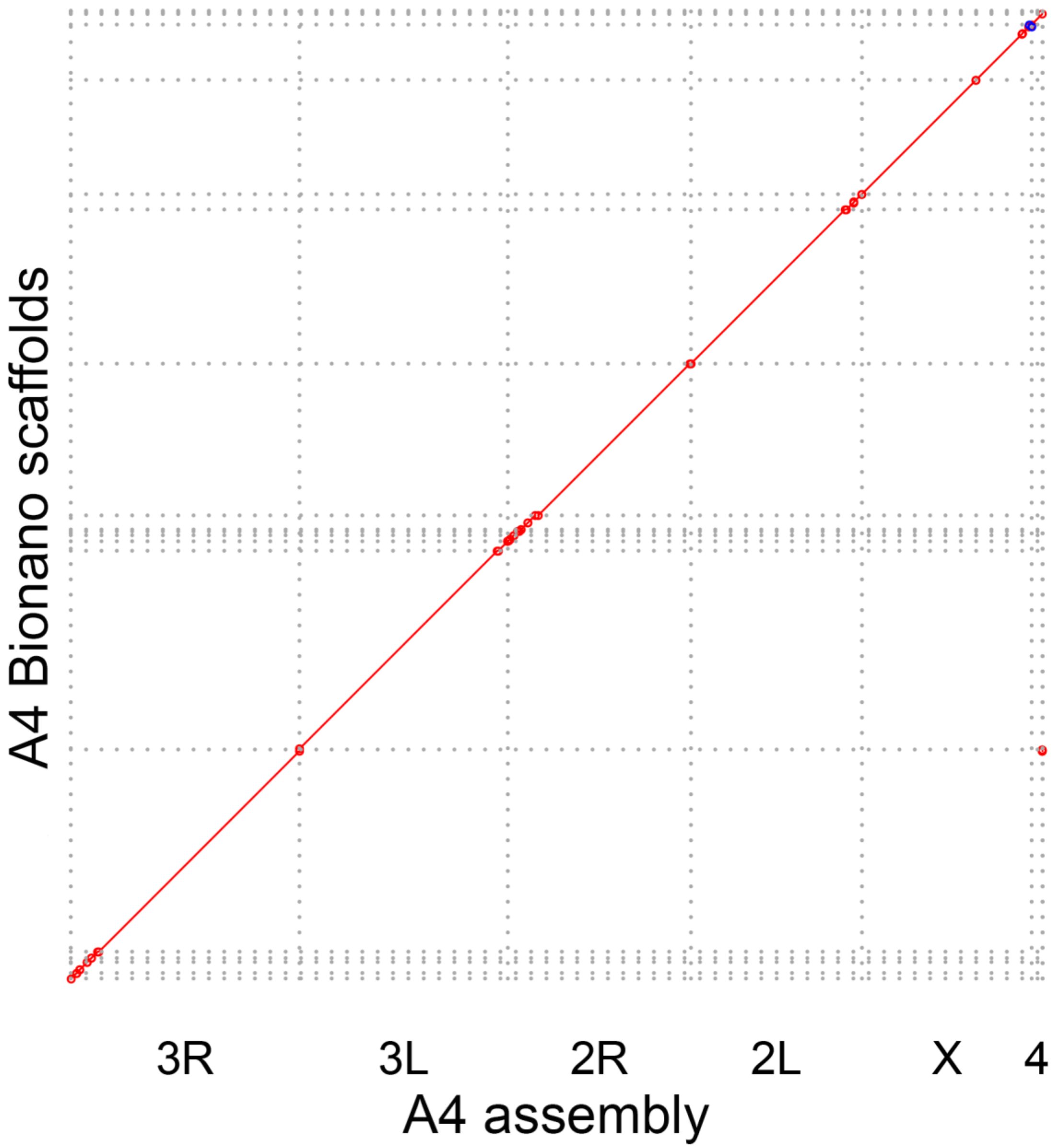

Supplementary Figure 4. Alignment dot plot between the A4 assembly scaffolded using the reference assembly[1] and an A4 assembly scaffolded with a Bionano optical map. Collinearity of the two assemblies suggest that the contiguity of A4 assembly is not a result of incorrect contig joining. Evidence of a strain specific inversion (blue) mapping to the distal end of the X chromosome is present in the Bionano assembly, but absent in the PacBio assembly.

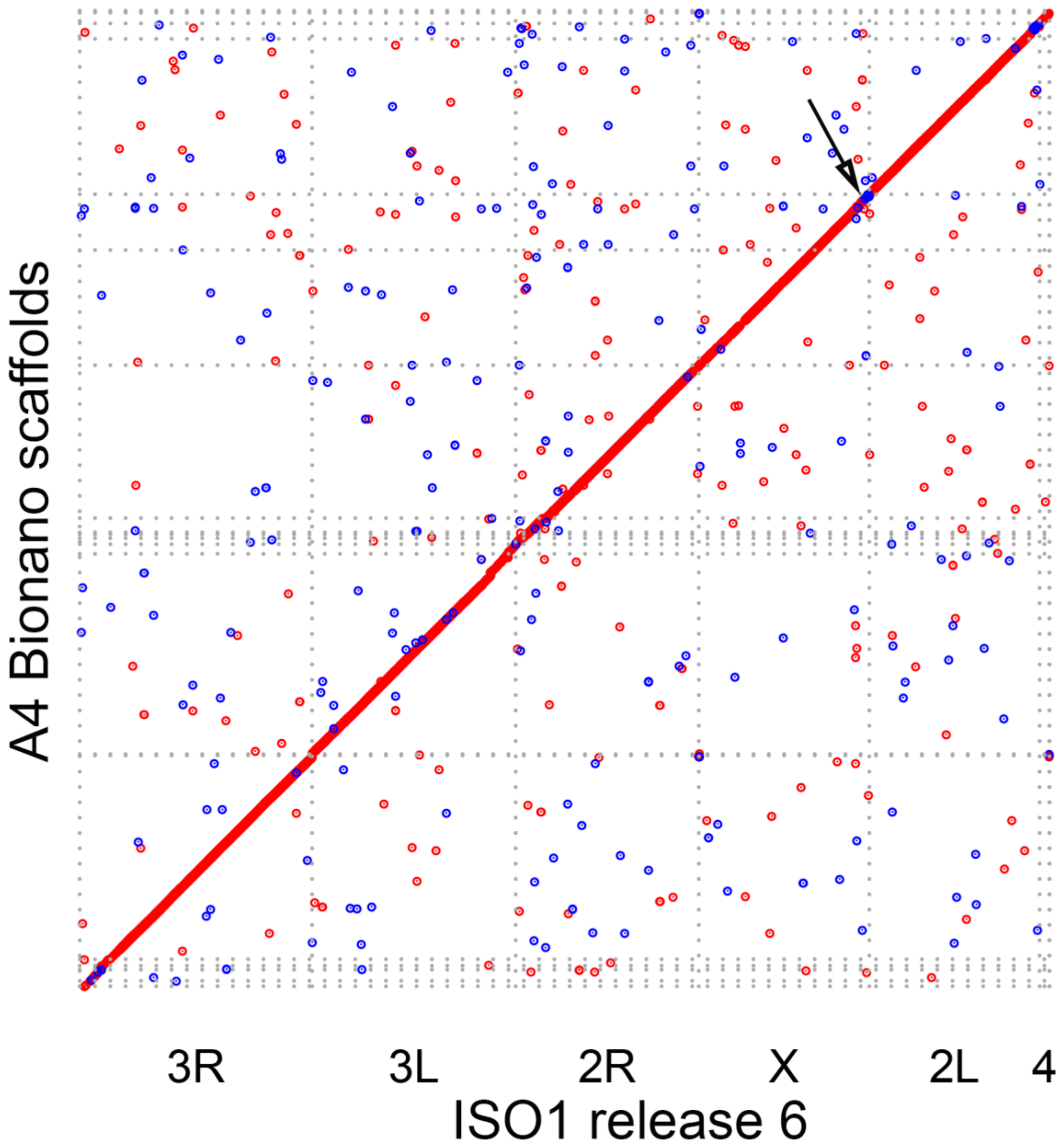

Supplementary Figure 5. Alignment dot plot between the ISO1 release 6 scaffolds and the A4 Bionano assembly scaffolds. The small off-diagonal alignments (dots) are due to TEs and repeat elements. The evidence of the X chromosome inversion (arrow) at the pericentric heterochromatin of A4 X chromosome is visible in a Bionano scaffold mapping to the distal end of ISO1 X.

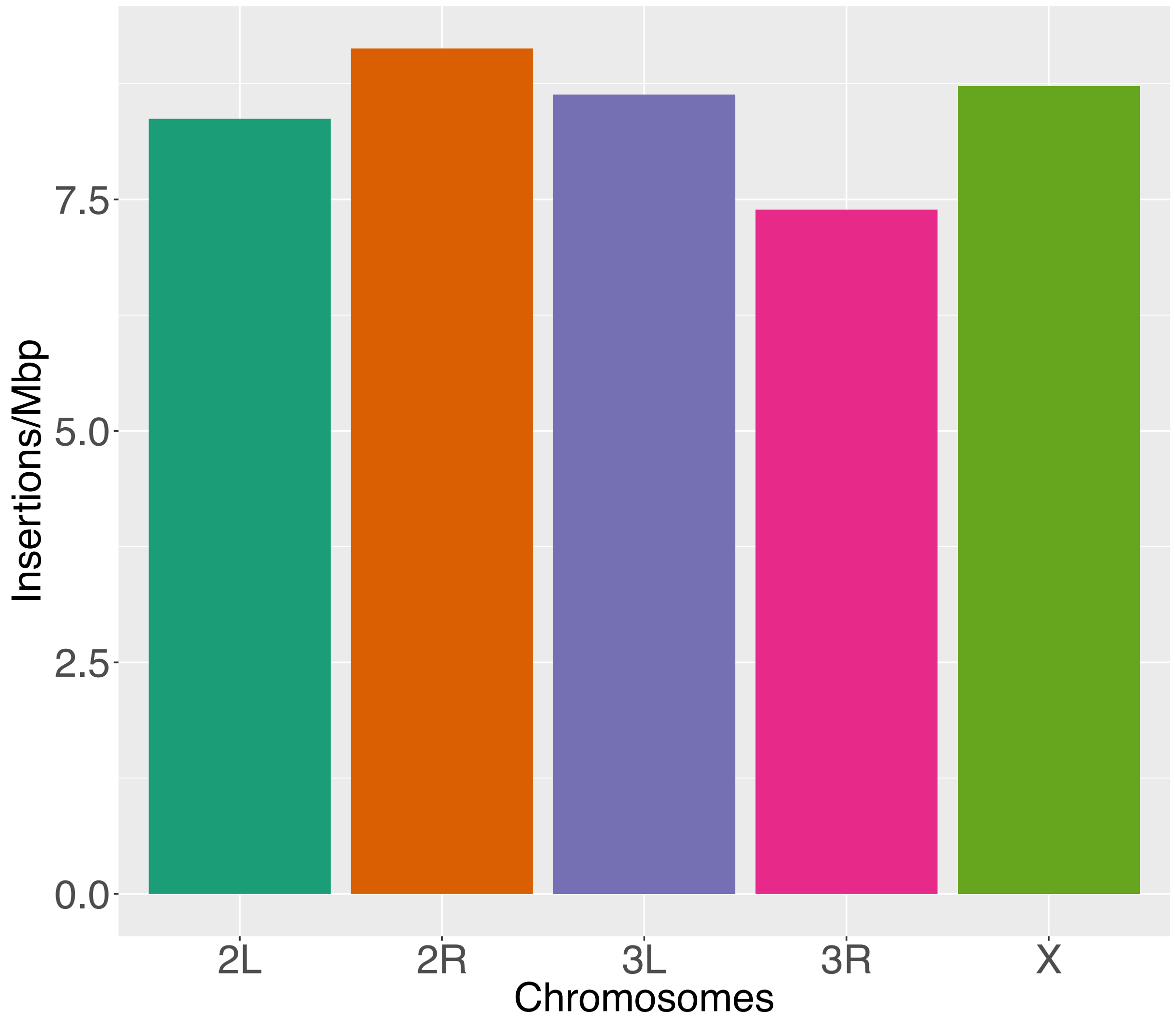

Supplementary Figure 6. Number of insertions per megabase of euchromatic DNA in each chromosome arm.

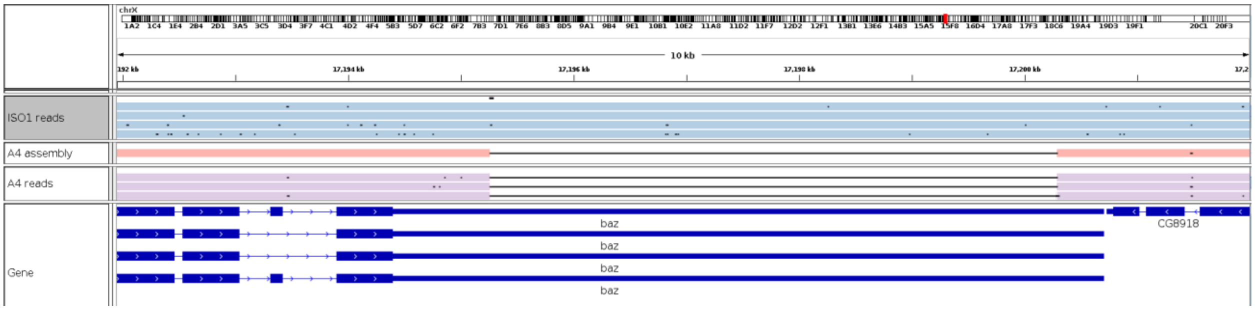

Supplementary Figure 7: Alignment tracks showing insertion of a TE in ISO1. The four tracks (top to bottom) represent the ISO1 assembly and ISO1 PacBio reads[2] (light blue), A4 assembly (orange), A4 PacBio reads[2] (purple) aligned to it. The alignment gap in the A4 assembly and the A4 long reads show that the TE known as *flea{}148* is absent in A4. The TE is inserted into the 3' UTR of the bazooka (*baz*) gene model[3].

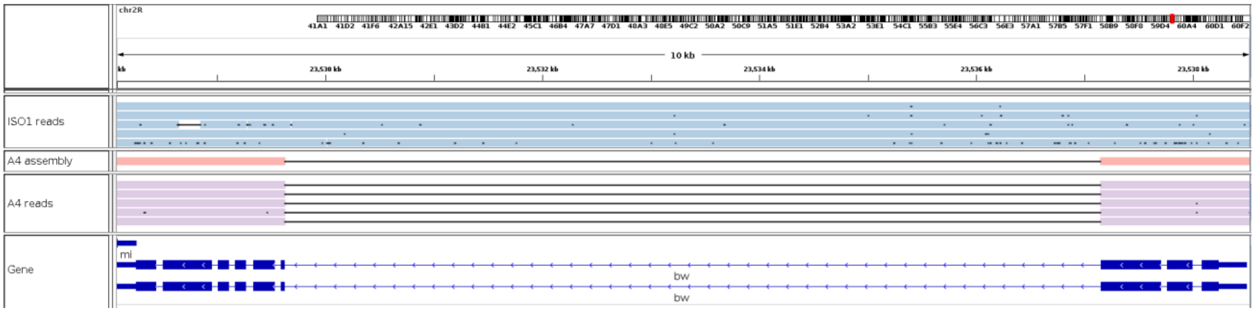

Supplementary Figure 8. Alignment tracks showing insertion of a TE in ISO1. The four tracks (top to bottom) represent the ISO1 assembly and ISO1 PacBio reads (light blue), A4 assembly (orange), A4 PacBio reads (purple) aligned to it. The alignment gap in the A4 assembly and the A4 long reads show that the TE known as *412{}bw* is absent in A4. The TE is inserted into an exon of the brown (*bw*) gene and likely disrupts the function of the gene to cause a visible change in eye color[4].

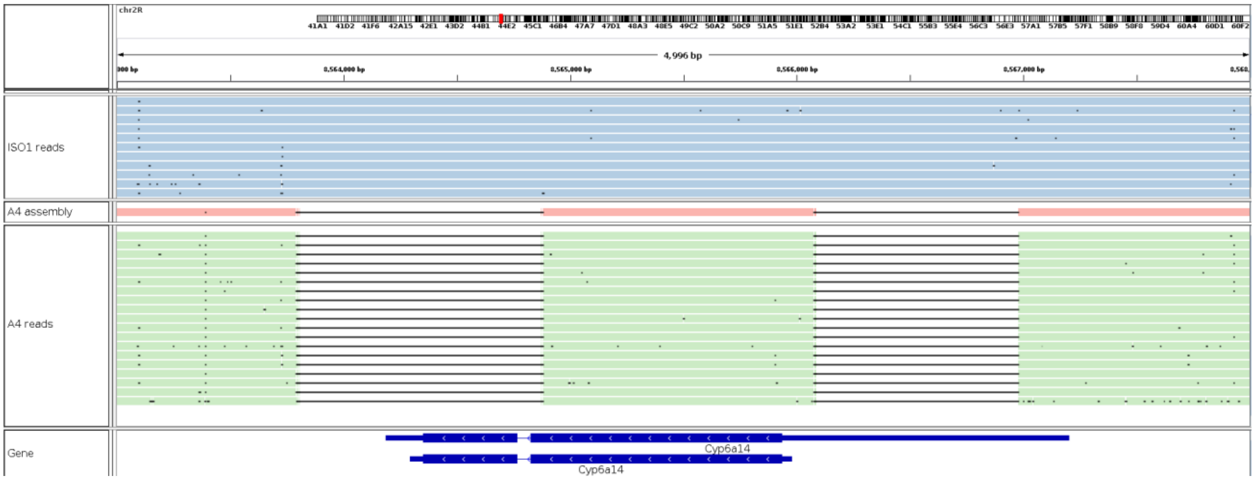

Supplementary Figure 9. Alignment tracks showing partial deletion of the gene *Cyp6a14* and insertion of a TE in the 5' UTR of the gene in ISO1. The four tracks (top to bottom) represent the ISO1 assembly and ISO1 PacBio reads (light blue), A4 assembly (orange), A4 PacBio reads (green) aligned to it. The alignment gap in the A4 assembly and the A4 long reads show that the second exon and 3' UTR of *Cyp6a14* (left) and a TE known as *1360{}780* (right) are absent in A4. The TE is inserted into the 5' UTR of *Cyp6a14*[3].

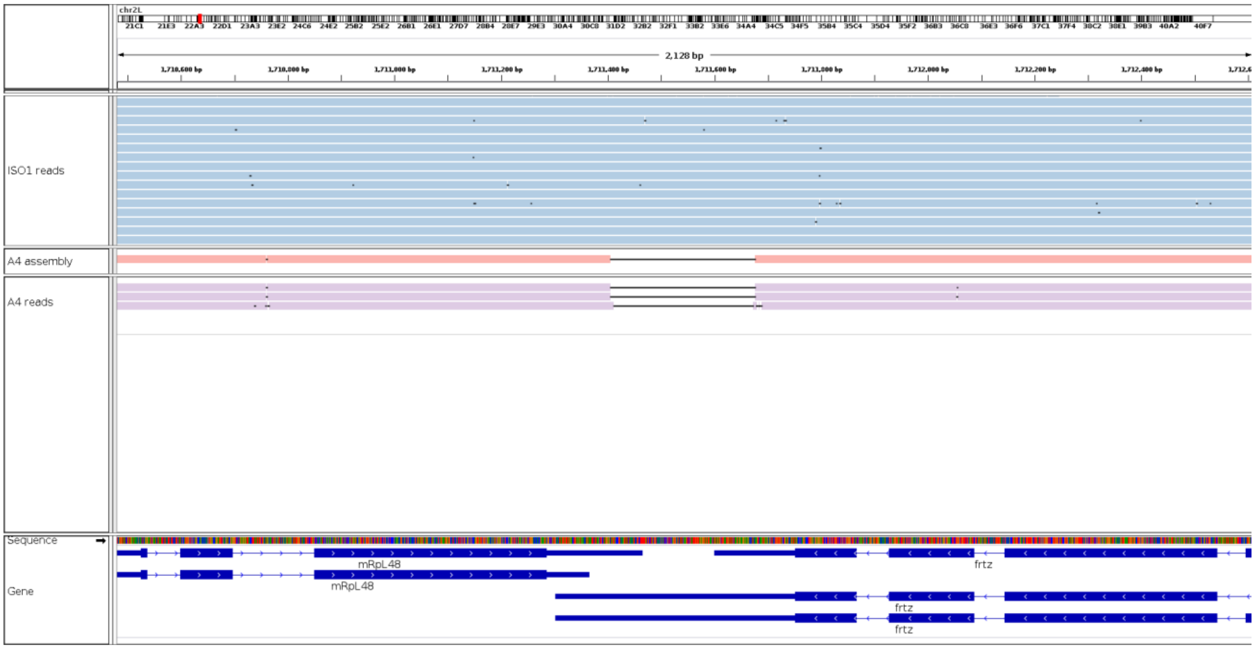

Supplementary Figure 10. Alignment tracks showing insertion of a TE in ISO1. The four tracks (top to bottom) represent the ISO1 assembly and ISO1 PacBio reads (light blue), A4 assembly (orange), A4 PacBio reads (purple) aligned to it. The alignment gap in the A4 assembly and the A4 long reads show that the TE known as *mdg3{}290* is absent in A4. The TE is inserted into the 3' UTRs of *mRpL48* and fritz (*frtz*) and increases the lengths of the 3' UTR for both genes[3].

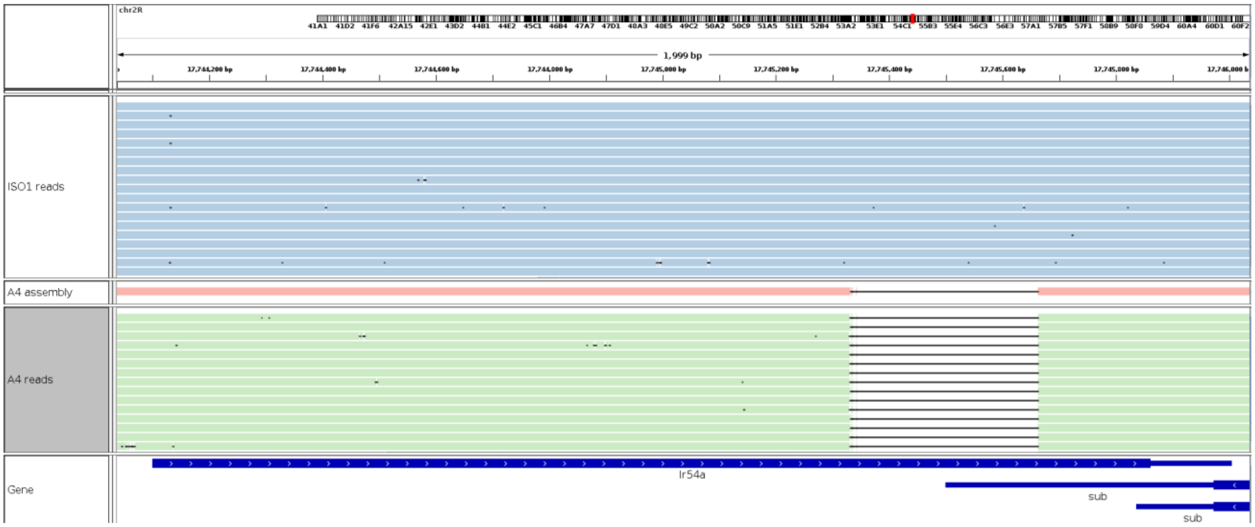

Supplementary Figure 11. Alignment tracks showing partial deletion of Ionotropic receptor 54a (*Ir54a*) and subito (*sub*) in A4. The four tracks (top to bottom) represent the ISO1 assembly and ISO1 PacBio reads (light blue), A4 assembly (orange), A4 PacBio reads (green) aligned to it. The alignment gap in the A4 assembly and the A4 long reads show that part of the *Ir54a* coding sequence and 3' UTR of *sub* is absent in A4.

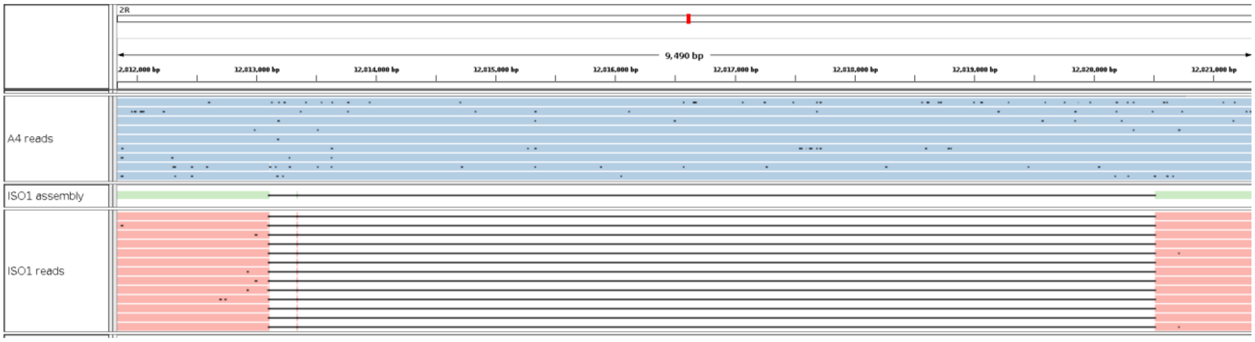

Supplementary Figure 12. Alignment tracks showing insertion of a TE in A4. The four tracks (top to bottom) represent the A4 assembly and A4 PacBio reads (light blue), ISO1 release 6 assembly (green), ISO1 PacBio reads (orange) aligned to it. The alignment gap in the ISO1 assembly and the ISO1 long reads show that the TE known as *blood* is inserted in A4 but absent in ISO1. The TE is inserted into the last intron of the gene Aspartyl-tRNA synthetase (*AspRS*) and 5' UTR/first intron of the gene N-methyl-D-aspartate receptor-associated protein (*Nmda1*)[3] (gene models not shown).

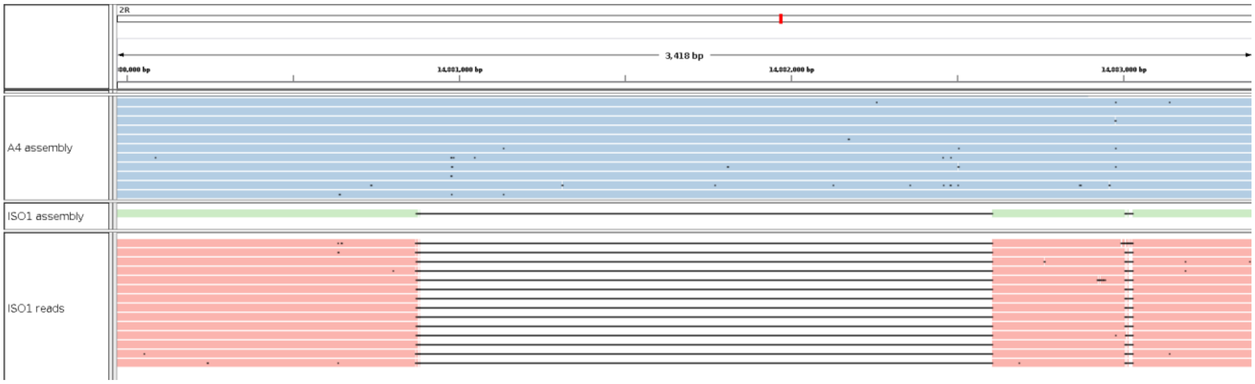

Supplementary Figure 13. Alignment tracks showing insertion of a TE in A4. The four tracks (top to bottom) represent the A4 assembly and A4 PacBio reads (light blue), ISO1 release 6 assembly (green), ISO1 PacBio reads (orange) aligned to it. The alignment gap in the ISO1 assembly and the ISO1 long reads show that an *S* element TE is inserted in A4 but absent in ISO1. The TE is inserted into the 3' UTR of the gene Cytochrome P450-6a9 (*Cyp6a9;* gene model not shown)[3].

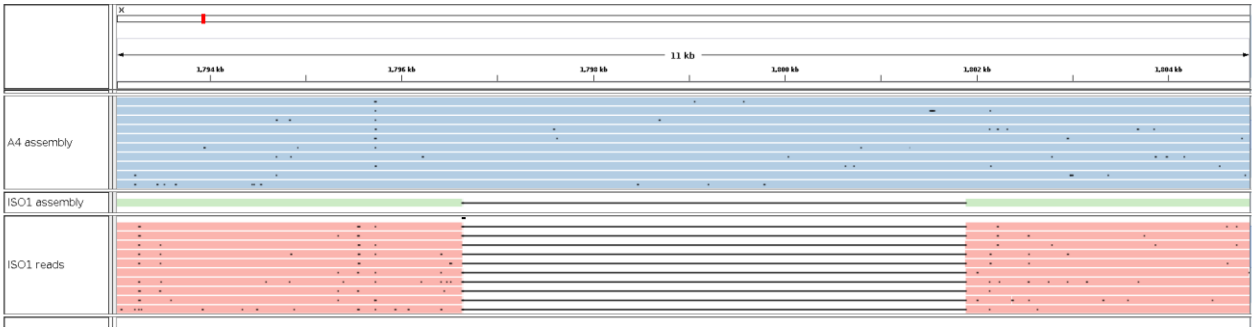

Supplementary Figure 14. Alignment tracks showing insertion of a TE in A4. The four tracks (top to bottom) represent the A4 assembly and A4 PacBio reads (light blue), ISO1 release 6 assembly (green), ISO1 PacBio reads (orange) aligned to it. The alignment gap in the ISO1 assembly and the ISO1 long reads show that a LTR retrotransposon known as *Transpac* is inserted in A4 but absent in ISO1. The TE is inserted into the 3' UTR of the gene ER degradation enhancer, mannosidase alpha-like 1 (*Edem1*; not shown).

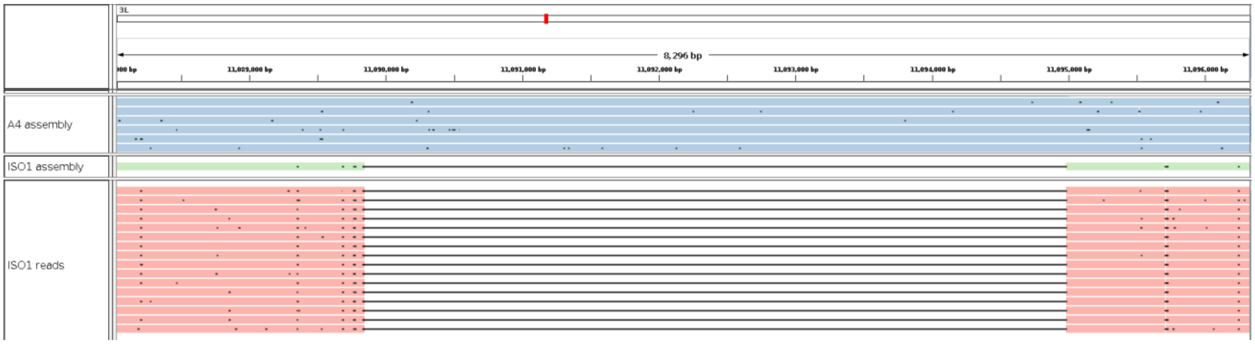

Supplementary Figure 15. Alignment tracks showing insertion of a TE in A4. The four tracks (top to bottom) represent the A4 assembly and A4 PacBio reads (light blue), ISO1 release 6 assembly (green), ISO1 PacBio reads (orange) aligned to it. The alignment gap in the ISO1 assembly and the ISO1 long reads show that a *Copia* LTR transposon is inserted in A4 but absent in ISO1. The TE is inserted into the 3' UTR of the gene tonalli (*tna*; not shown).

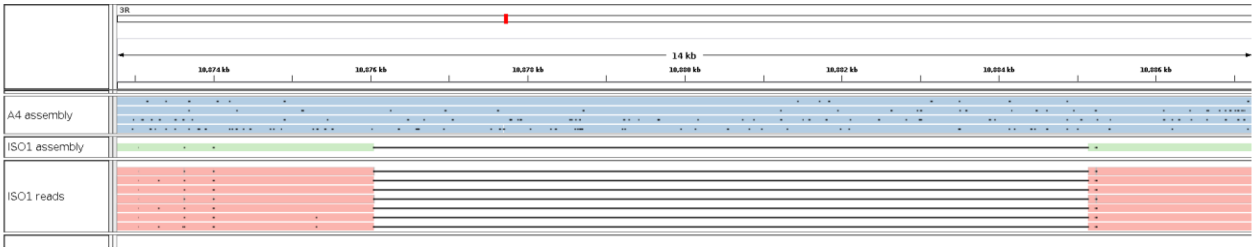

Supplementary Figure 16. Alignment tracks showing insertion of a TE in A4. The four tracks (top to bottom) represent the A4 assembly and A4 PacBio reads (light blue), ISO1 release 6 assembly (green), ISO1 PacBio reads (orange) aligned to it. The alignment gap in the ISO1 assembly and the ISO1 long reads show that a *Mdg3* LTR transposon is inserted in A4 but absent in ISO1. The TE is inserted into the 3’ UTR of the gene *Ugt86Di* (not shown) that encodes a UDP-glucosyltransferase enzyme.

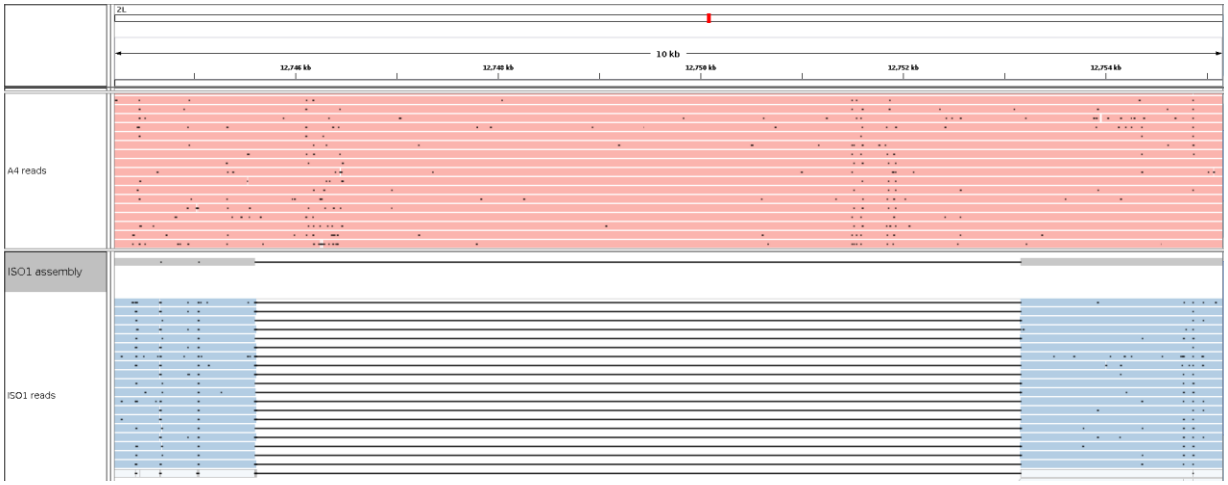

Supplementary Figure 17. A TE insertion that is absent in the list of A4 TE insertion genotypes based on paired end Illumina reads[5,6]. The four tracks (top to bottom) represent the A4 assembly and A4 PacBio reads (orange), ISO1 assembly (grey), ISO1 PacBio reads (light blue) aligned to it. The insertion is located right next to an existing TE called INE-12210 inside an intron of the gene *MRP* (*Multidrug-Resistance like Protein 1*). Such existing TEs obscure the TE insertion signals that short-read based methods[5] use to detect TE insertions.

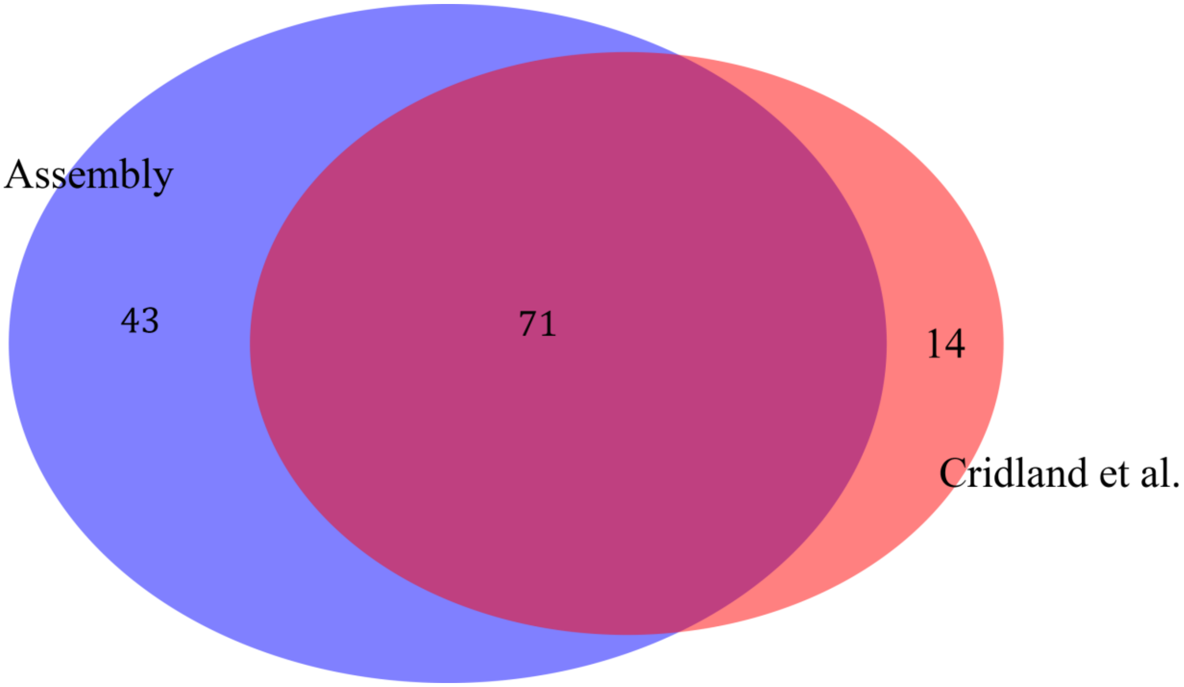

Supplementary Figure 18. The short-read based TE insertion genotyping method of [6] detects 63% of TE insertions present in the euchromatic regions of the 2L chromosome arm of A4 assembly. All TE calls for all categories above were manually curated. The 37% of TEs missed are considered hidden/invisible to short-read methods.

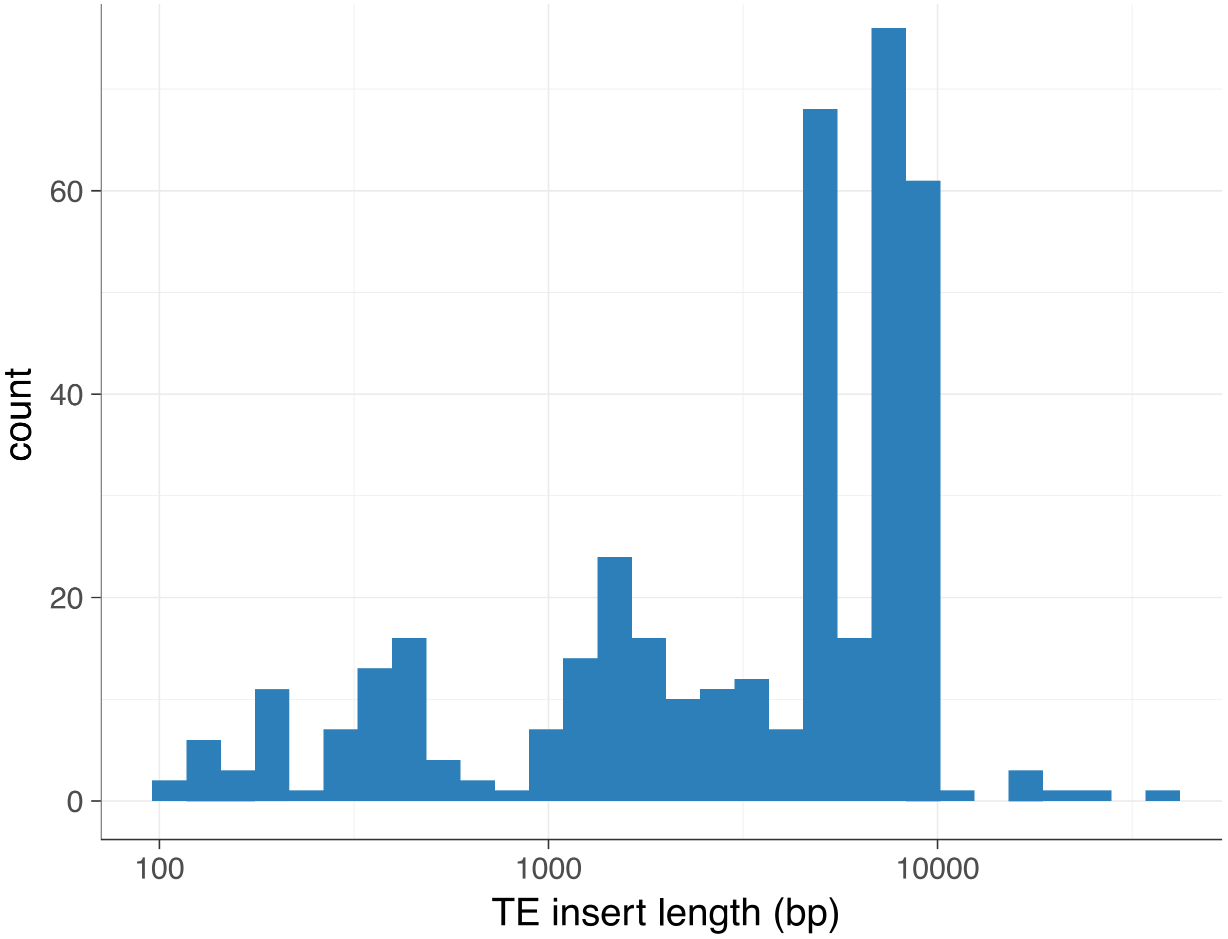

Supplementary Figure 19. The distribution of lengths of the TEs that insert within introns. More than 50% TEs inserting within introns are large (>5 kbp; median 5,016 bp) and may cause 'intron delay'[8].

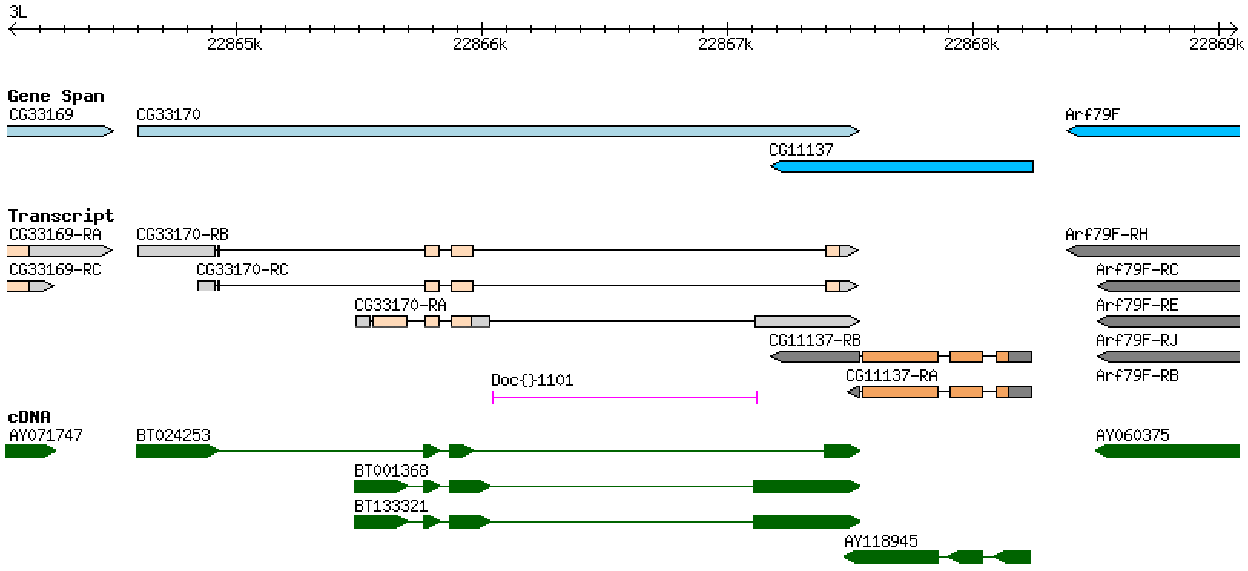

Supplementary Figure 20. Insertion of a private TE (*Doc* element) in the ISO1 gene *CG33170* corresponds to the whole fourth intron. Isoforms are shown as FlyBase[3] gene annotations. The cDNA annotations are based on Drosophila Gene Collection[9].

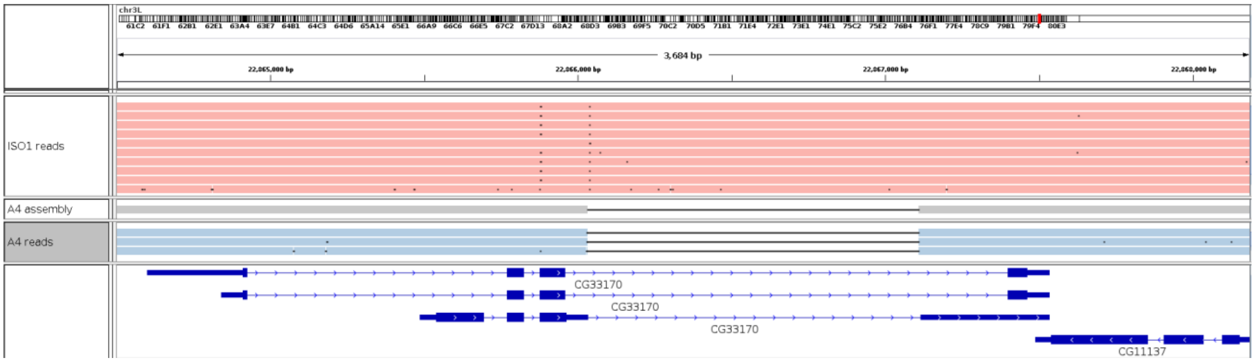

Supplementary Figure 21. The A4 long reads (light blue) and A4 assembly (grey) aligned to the ISO1 release 6 assembly to show the ISO1 specific *Doc* element insertion (Supplementary Fig. 20) in the gene *CG33170*. As demonstrated by the schematic diagrams (blue lines and rectangles) of the *CG33170* transcripts, this private TE in ISO1 covers the entire intron in one of the transcript isoforms of *CG33170*.

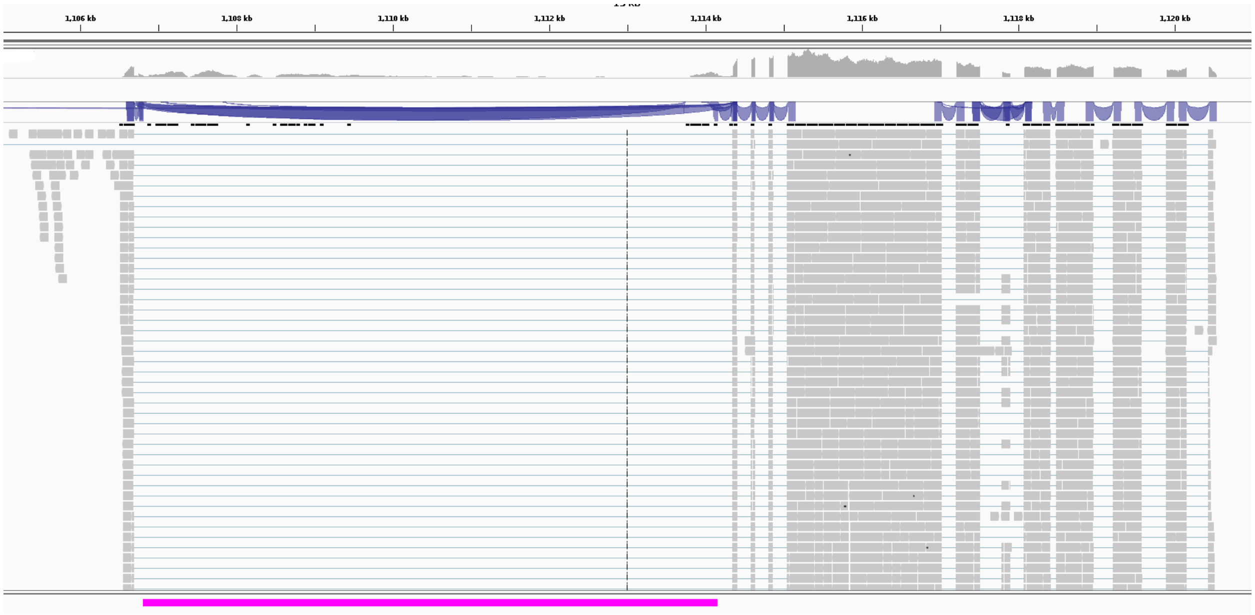

Supplementary Figure 22. Example of a new intron in *CG13900* of A4 gained via *Mdg1* LTR insertion (the pink bar). A4 RNA-seq reads[7] were mapped to the A4 assembly using *TopHat* (Online Methods). The blue lines and the purple ribbons indicate intron and splice junctions. For this TE intron, individual reads span the entire 7,348 bp insertion, suggesting that the TE is spliced out. Grey rectangles represent reads.

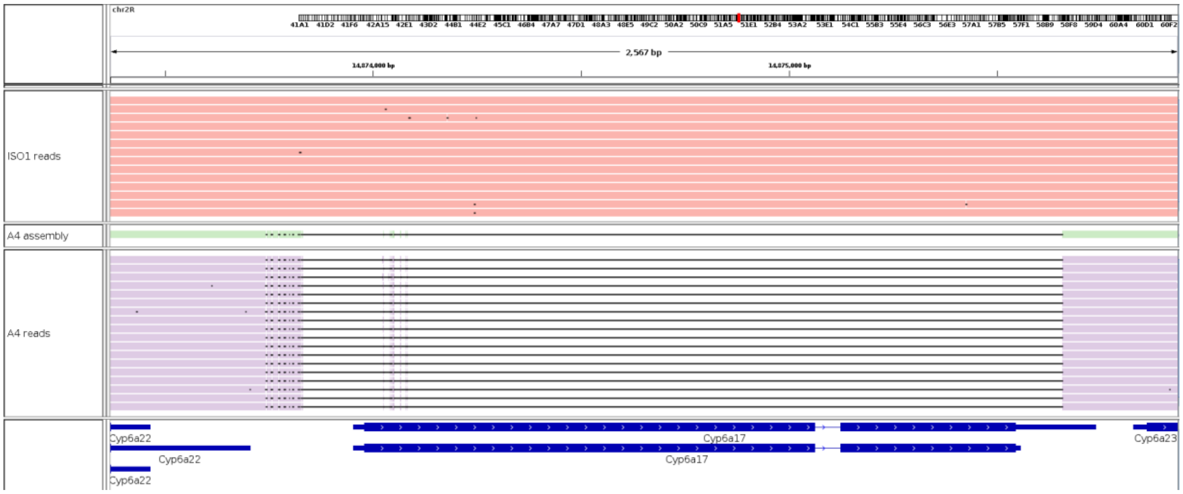

Supplementary Figure 23. Alignment tracks showing complete deletion of *Cytochrome P450-6a17* (*Cyp6a17*) in A4. The four tracks (top to bottom) represent the ISO1 assembly and ISO1 PacBio reads (light blue), A4 assembly (orange), A4 PacBio reads (green) aligned to it. The alignment gap in the A4 assembly and the A4 long reads show that the entire *Cyp6a17* is absent in A4.

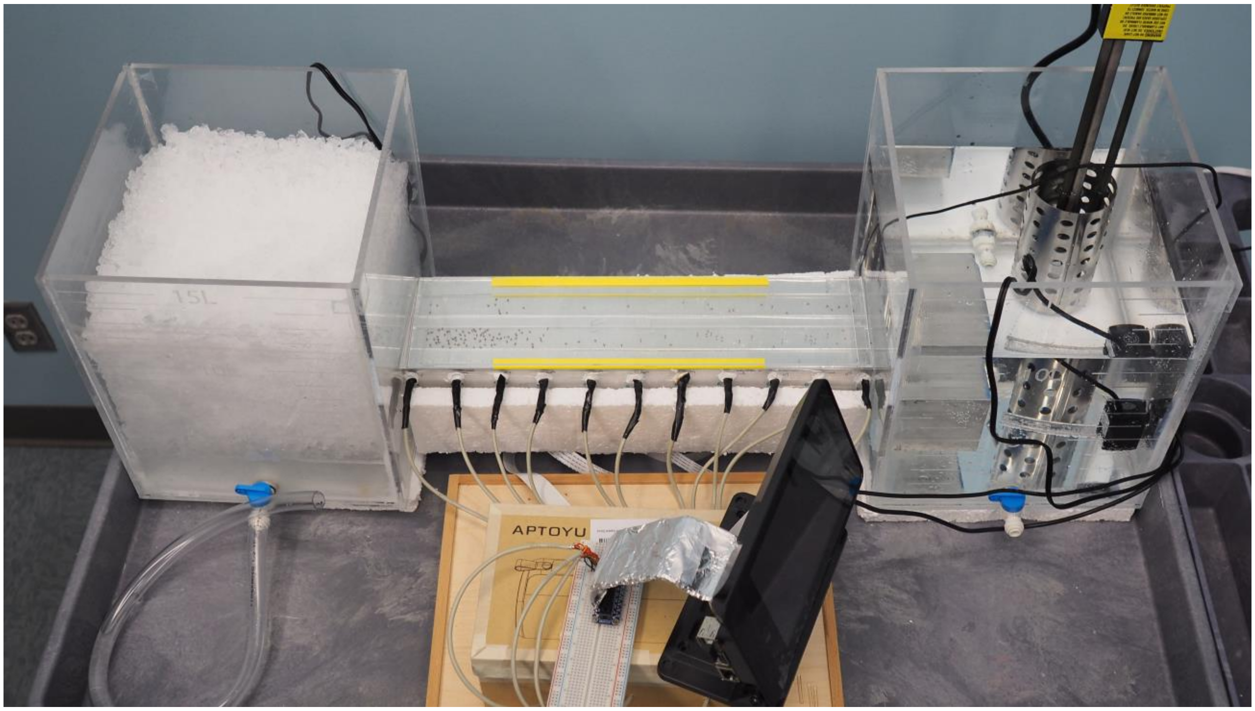

Supplementary Figure 24. The temperature preference assay apparatus. The two ends of the aluminum block are inserted into a cold (0°C) and a warm water (35°C) reservoir. Temperature of the warm water was maintained using a water heater which was connected to a temperature controlled thermostat. Temperatures along the block were measured using thermal probes embedded in the bar. Temperature data was acquired with a custom Python script (see URLs) running on a Raspberry Pi (see Online Methods).

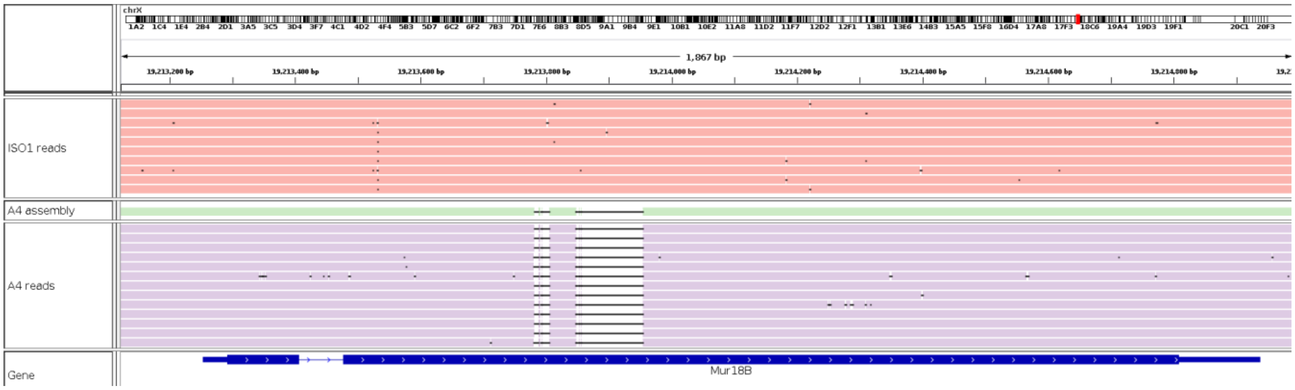

Supplementary Figure 25. Alignment tracks showing partial deletion of the Mucin related 18B gene (*Mur18B*) in A4. The four tracks (top to bottom) represent ISO1 assembly and ISO1 PacBio reads (orange), A4 assembly (green), A4 PacBio reads (purple) aligned to it. The alignment gap in the A4 assembly and the A4 long reads show that part of the *Mur18B* coding sequence (129bp) is deleted in A4.

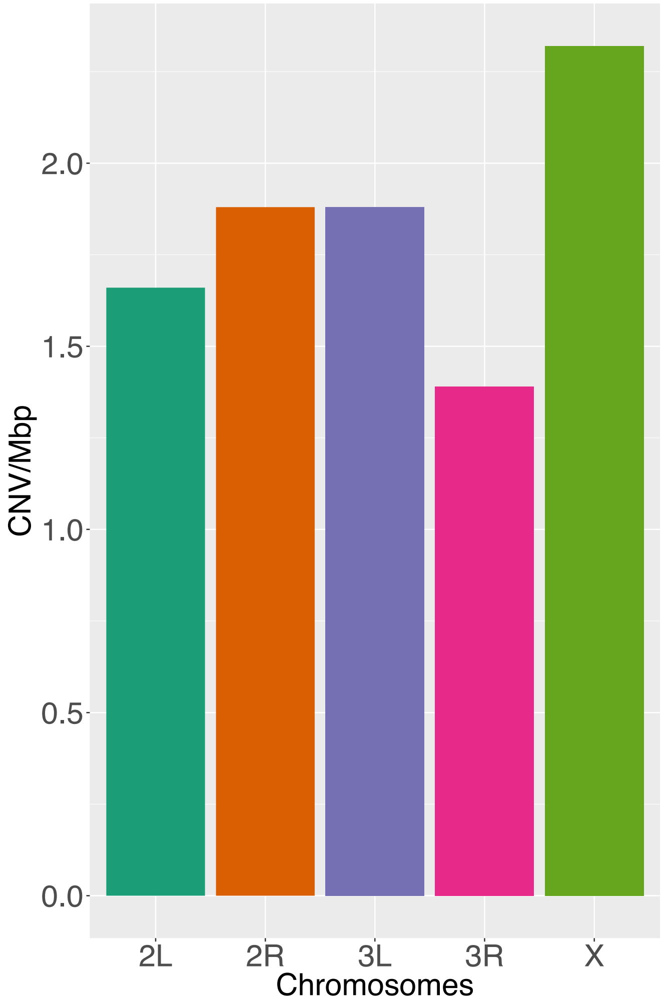

Supplementary Figure 26. Number of duplications per megabase of euchromatin.

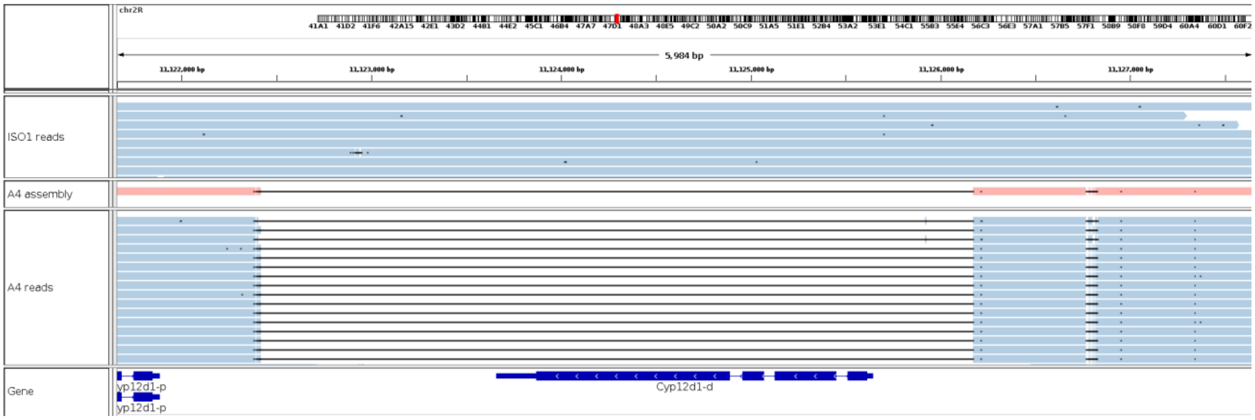

Supplementary Figure 27. Alignment tracks showing absence of Cytochrome P450 12d1-d (*Cyp12d1-d*) in A4. The four tracks (top to bottom) represent the ISO1 assembly and ISO1 PacBio reads (light blue), A4 assembly (orange), A4 PacBio reads (light blue) aligned to it. The missing sequence in A4 is duplicated in ISO1 and the alignment gap is due to the missing copy of in A4. The *Cyp12d1* gene overlaps a caffeine resistance QTL[10] and the duplication is a strong candidate mutation for caffeine resistance.

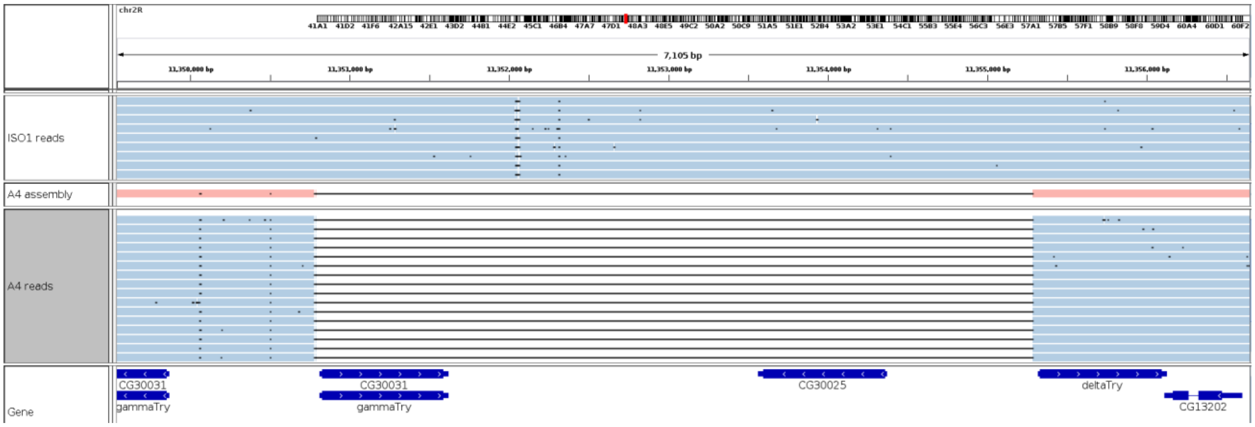

Supplementary Figure 28. Alignment tracks showing absence of a γTrypsin (γ*Try*) copy in A4. The four tracks (top to bottom) represent the ISO1 assembly and ISO1 PacBio reads (light blue), A4 assembly (orange), A4 PacBio reads (light blue) aligned to it. The missing sequence in A4 is duplicated in ISO1 and the alignment gap is due to the missing copy of the sequence in A4.

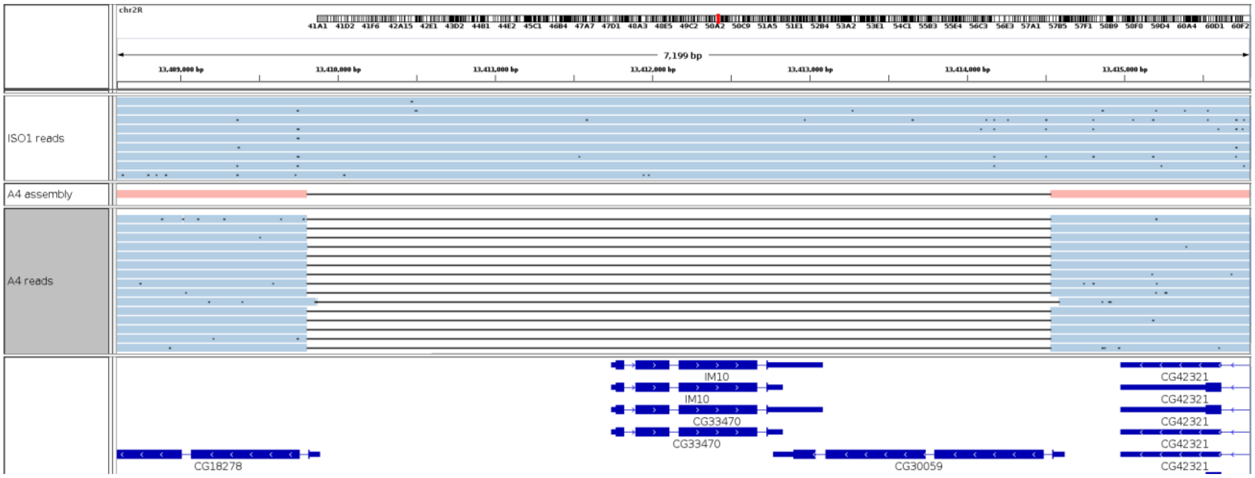

Supplementary Figure 29. Alignment tracks showing absence of Immune induced molecule prepropeptide (*IM10*) in A4. The four tracks (top to bottom) represent the ISO1 assembly and ISO1 PacBio reads (light blue), A4 assembly (orange), A4 PacBio reads (light blue) aligned to it. The missing sequence in A4 is duplicated in ISO1 and the alignment gap is due to the missing copy of the sequence in A4.

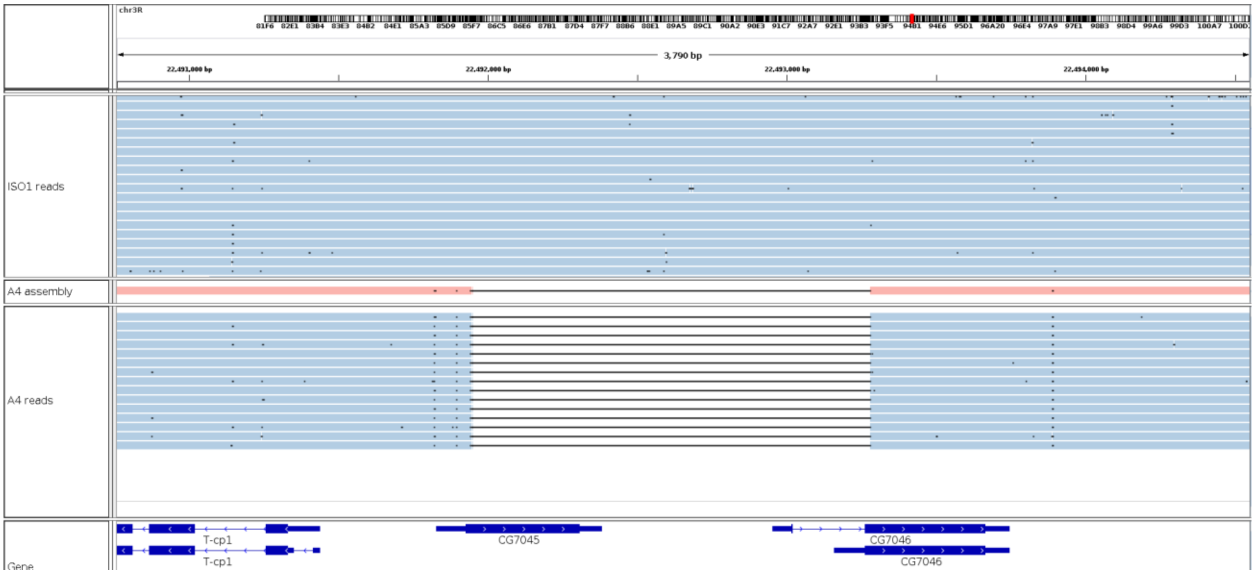

Supplementary Figure 30. Alignment tracks showing absence of testis-enriched HMG-box-containing protein2 (*tHMG2* or *CG7046*) in A4. The four tracks (top to bottom) represent the ISO1 assembly and ISO1 PacBio reads (light blue), A4 assembly (orange), A4 PacBio reads (light blue) aligned to it. The missing sequence in A4 is duplicated in ISO1 and the alignment gap is due to the missing copy of the sequence in A4.

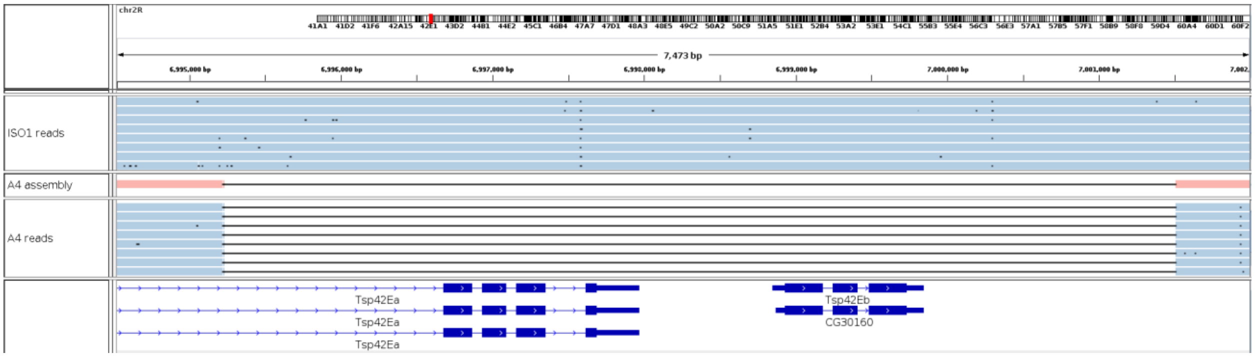

Supplementary Figure 31. Alignment tracks showing absence of part of Tetraspanin 42Ea (*Tsp42Ea*) and complete Tetraspanin 42Eb (*Tsp42Eb*) in A4. The four tracks (top to bottom) represent the ISO1 assembly and ISO1 PacBio reads (light blue), A4 assembly (orange), A4 PacBio reads (light blue) aligned to it. The missing sequence in A4 is duplicated in ISO1 and the alignment gap is due to the missing copy of the sequence in A4.

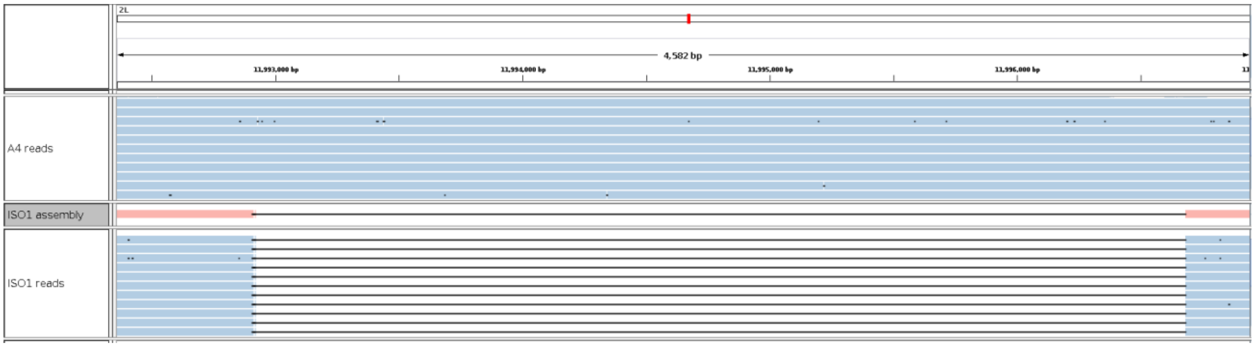

Supplementary Figure 32. Alignment tracks showing absence of a transcriptional Adaptor 1 (*Ada 1*; not shown) copy in ISO1. The four tracks (top to bottom) represent A4 assembly and A4 PacBio reads (light blue), ISO1 assembly (orange), ISO1 PacBio reads (light blue) aligned to it. The missing sequence in ISO1 is duplicated in A4 and the alignment gap is due to the missing copy of the sequence in A4.

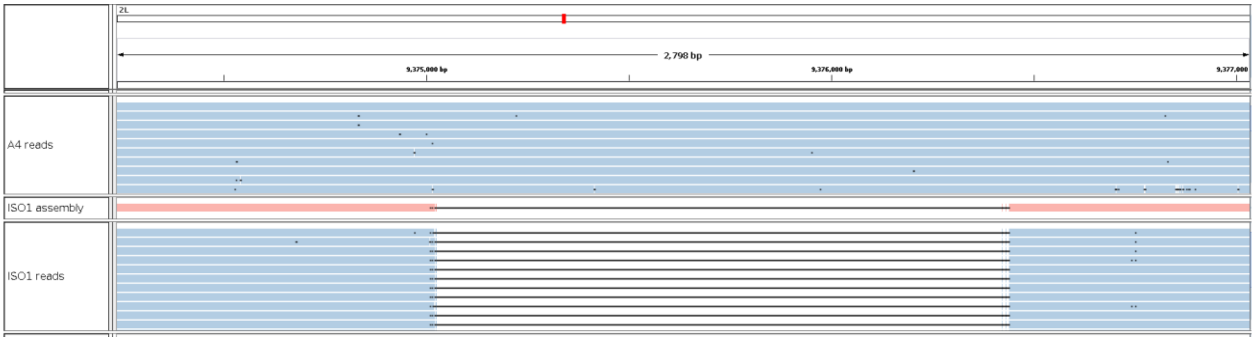

Supplementary Figure 33. Alignment tracks showing absence of a 1,382 bp intronic duplication in Aldehyde dehydrogenase (*Aldh*) gene in ISO1. The four tracks (top to bottom) represent the A4 assembly and A4 PacBio reads (light blue), ISO1 assembly (orange), ISO1 PacBio reads (light blue) aligned to it. The missing sequence in ISO1 is duplicated in A4 and the alignment gap is due to the missing copy of the sequence in A4.

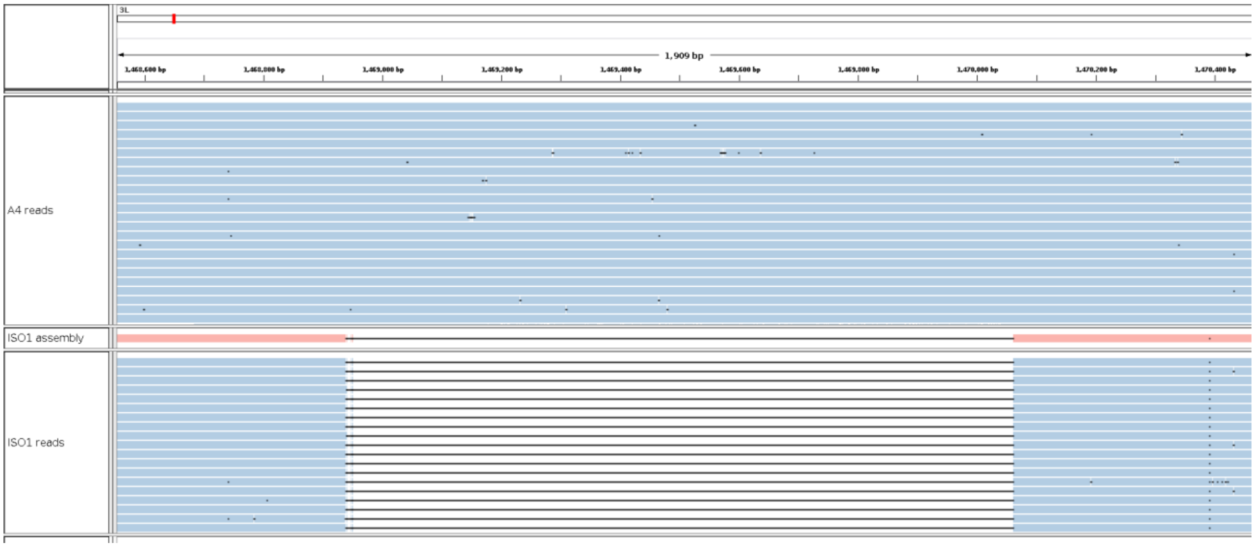

Supplementary Figure 34. Alignment tracks showing absence of Lysozyme D (*LysD*) gene copy in ISO1. The four tracks (top to bottom) represent theA4 assembly and A4 PacBio reads (light blue), ISO1 assembly (orange), ISO1 PacBio reads (light blue) aligned to it. The missing sequence in ISO1 is duplicated in A4 and the alignment gap is due to the missing copy of the sequence in A4. *LysD* expression is upregulated in A4 and the duplicate is a strong candidate mutation for upregulation.

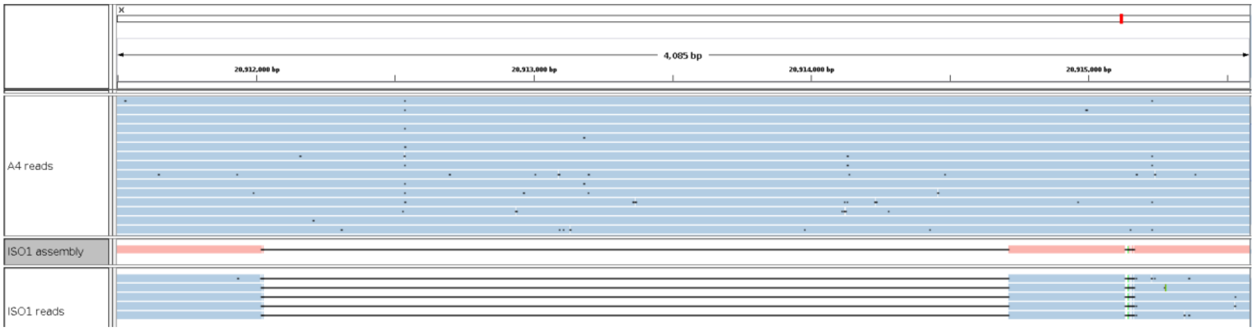

Supplementary Figure 35. Alignment tracks showing absence of a Microsomal glutathione S-transferase-like (*Mgstl*) gene copy in ISO1. The four tracks (top to bottom) represent the A4 assembly and A4 PacBio reads (light blue), ISO1 assembly (orange), ISO1 PacBio reads (light blue) aligned to it. The missing sequence in ISO1 is duplicated in A4 and the alignment gap is due to the missing copy of the sequence in A4. *Mgstl* expression is upregulated in A4 and the duplicate is a candidate mutation for upregulation.

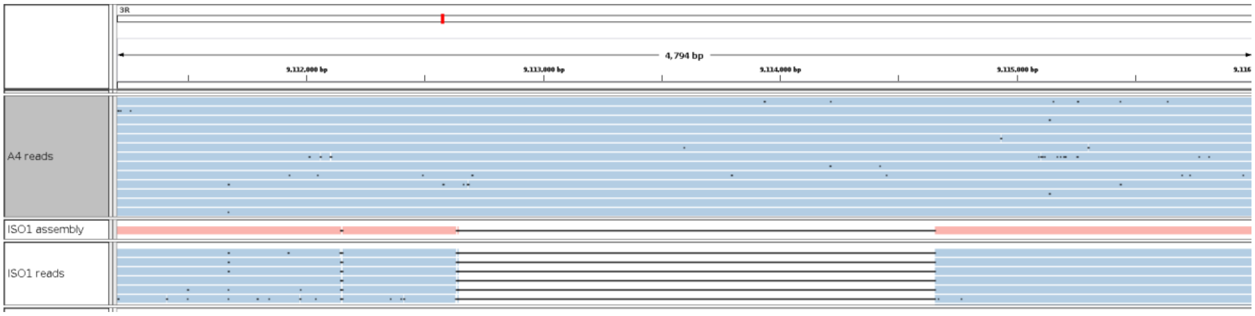

Supplementary Figure 36. Alignment tracks showing absence of a Odorant receptor 85f (*Or85f*) gene copy in ISO1. The four tracks (top to bottom) represent the A4 assembly and A4 PacBio reads (light blue), ISO1 assembly (orange), ISO1 PacBio reads (light blue) aligned to it. The missing sequence in ISO1 is duplicated in A4 and the alignment gap is due to the missing copy of the sequence in A4. *Or85f* expression is upregulated in A4 and the duplicate is a candidate mutation for upregulation.

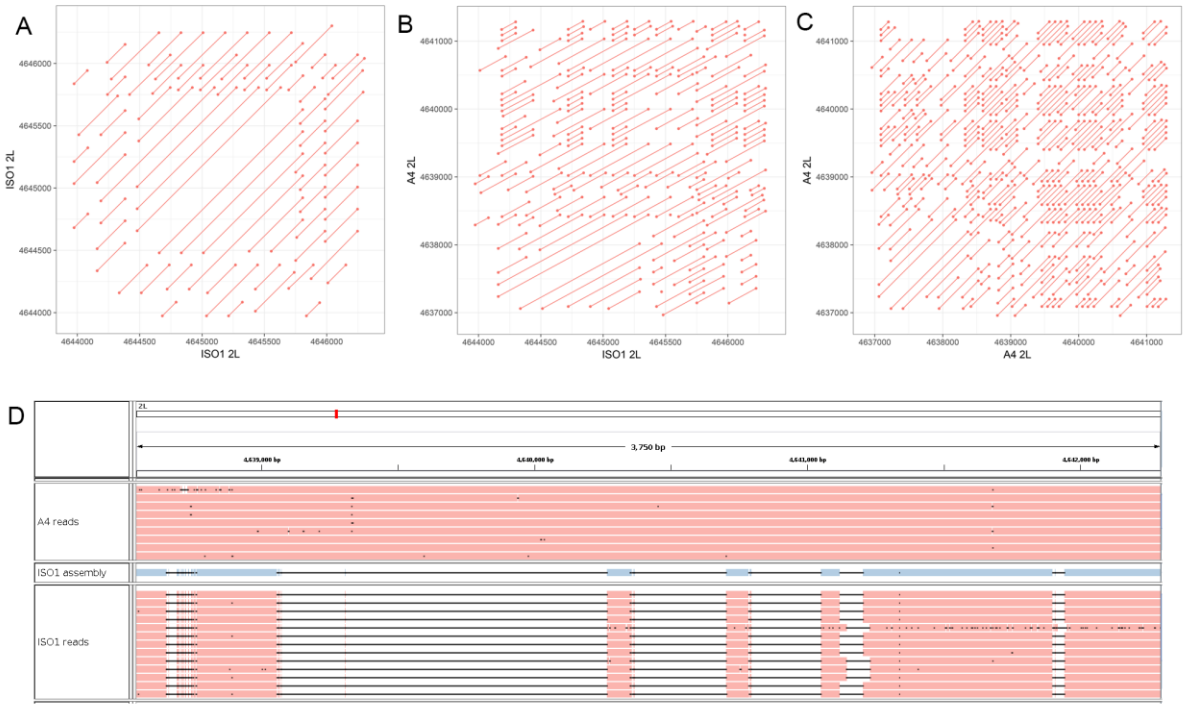

Supplementary Figure 37. Superior assembly of repeat-rich regions using PacBio reads. A) Alignment dot plot between a region in the 3rd exon of the gene *CG15635* in ISO1 (X-axis) 2L chromosome arm with itself. **B)** Alignment dot plot between the same region in A and the corresponding region in the A4 2L chromosome arm. The alignment gaps in the ISO1 assembly and reads are due to the fewer repeats in ISO1. **C)** Alignment dot plot between the A4 sequence in B and itself. Each line in the dot plots represents an alignment between the corresponding genomic regions in X and Y, and the dots represent the beginning and end of each of these alignments. The dot plot shows the complex structure of the region, which consists of multiple overlapping repeats. A4 has more copies of a repeat unit. The A4 reads show contiguous alignment with the A4 assembly at this region, showing that the CNV is not due to assembly error.

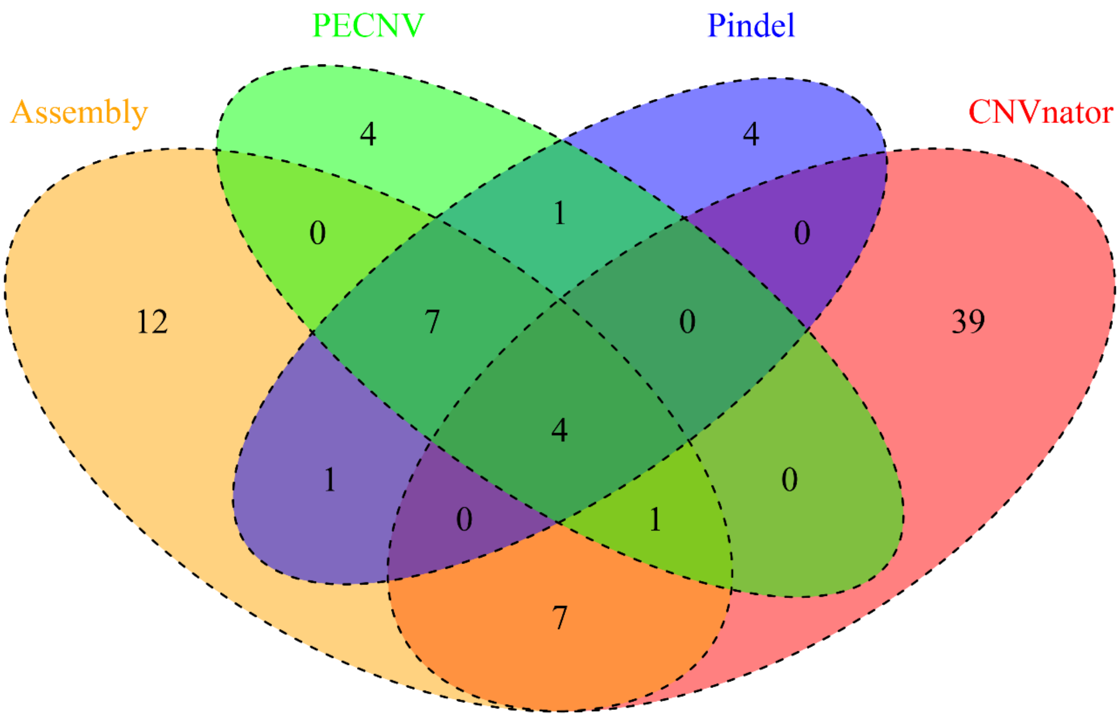

Supplementary Figure 38. Tandem duplications detected by our A4-ISO1 genome alignment approach (Assembly), a split read mapping method (*Pindel*[11]), a read pair orientation method (*PECNV*[12]), and a read coverage based method (*CNVnator*[13]) on chromosome arm 2L. All mutation calls were manually curated (Supplementary Methods). Mutations detected by *PECNV* and *Pindel* overlap significantly, whereas CNVs detected by *CNVnator* overlap little with those detected by *Pindel* and *PECNV*. *CNVnator* calls, when not confirmed by other methods, are dominated by false positives and yield a similar false negative rate as the other methods. When used in concert with *PECNV*, it permits discovery of one additional mutation on 2L. Mutations discovered by any two methods were considered discoverable by short reads. Other mutations are considered hidden/invisible.

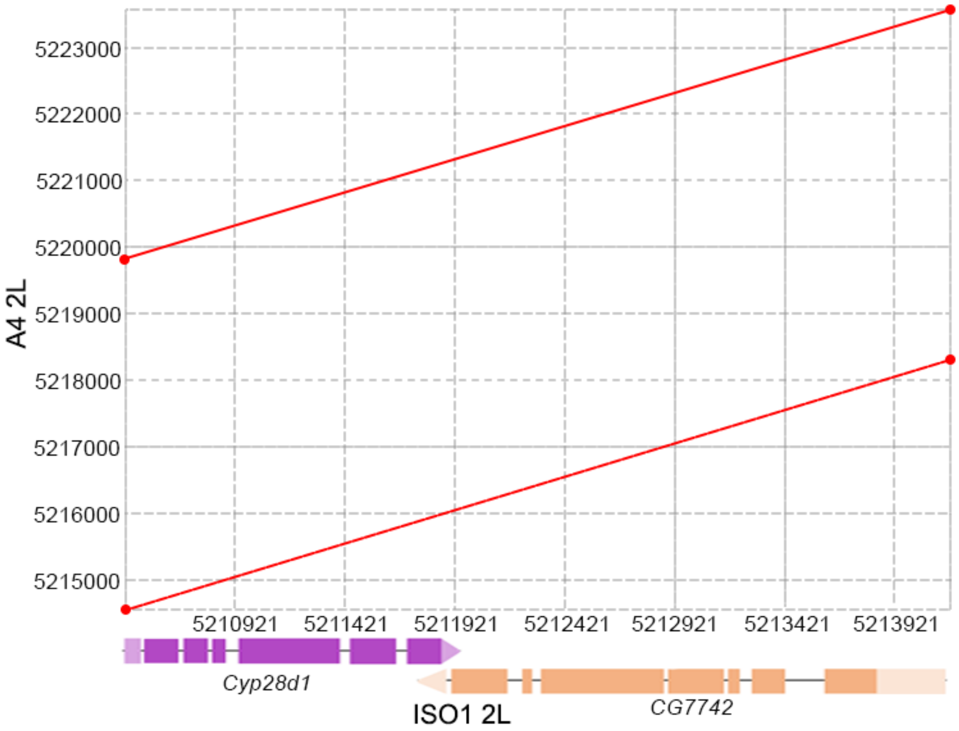

Supplementary Figure 39. Dot plot between the ISO1 2L segment (5,210,421-5,214,176) containing *Cyp28d1* and *CG7742* and the region 5,215,000-5,223,000 on chromosome arm 2L in A4 (Y axis). The duplicates are shown as the two parallel lines spanning the entire ISO1 segment. The vertical space between the end of the bottom red line and the beginning of the top red line is due to the 1.5 kbp fragment derived from an *Accord* element.

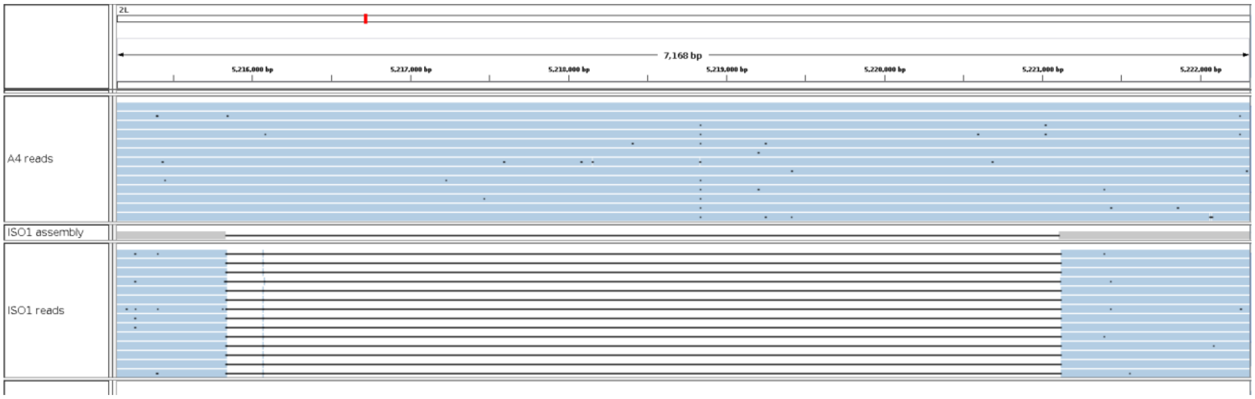

Supplementary Figure 40. Alignment of A4 long reads, ISO1 assembly, and ISO1 long reads to the A4 genomic region containing the two copies of *Cyp28d1*. The alignment gap in the ISO1 assembly and the ISO1 reads is due to absence of the second copy of *Cyp28d1* and the *Accord* insertion.

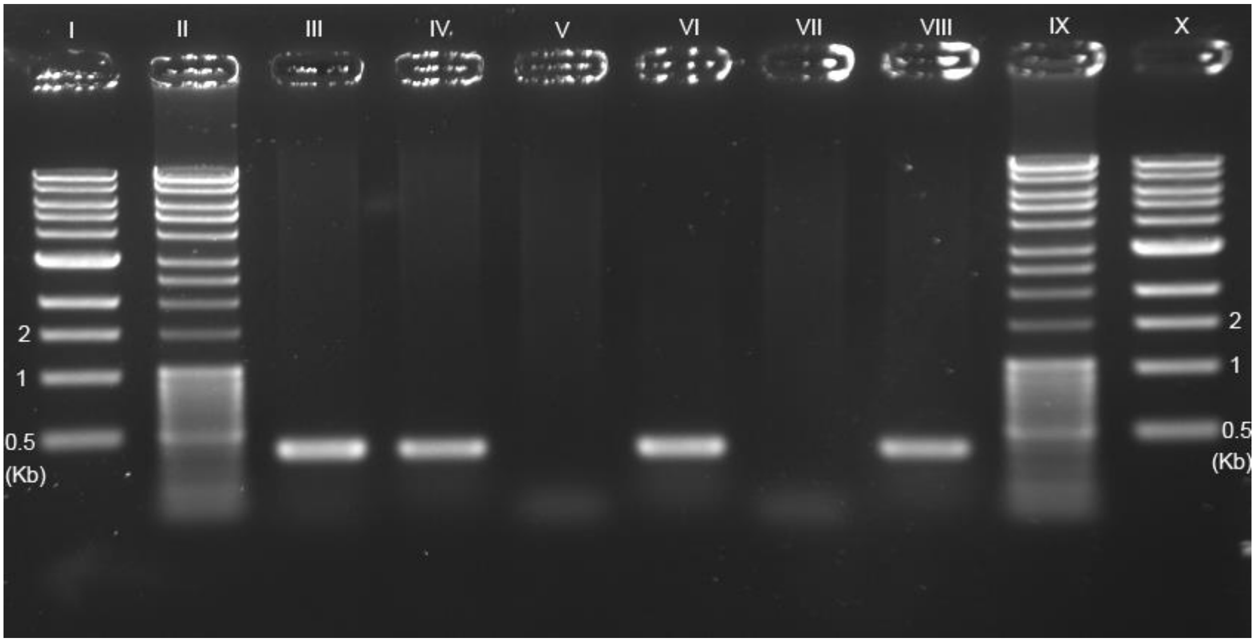

Supplementary Figure 41. Gel image showing presence and absence of *Cyp28d1* duplicates in A4, ISO1, and A3. From left to right: I. 1 Kb ladder from New England Biolabs; II. 2-Log ladder from New England Biolabs. III. A4 DNA amplified with *Cyp28d1* proximal copy specific primer (217) produces a 408 bp band; IV. A4 DNA amplified with *Cyp28d1* distal copy specific primer (222) produces a 443 bp band; V. ISO1 DNA amplified with primer 217; VI. ISO1 DNA amplified with primer 222. VII. A3 DNA amplified with primer 217. VIII. A3 DNA amplified with primer 222. IX and X are same as II and I, respectively. This gel shows that A4 has two copies of the gene, whereas ISO1 and A3 has one copy of the gene.

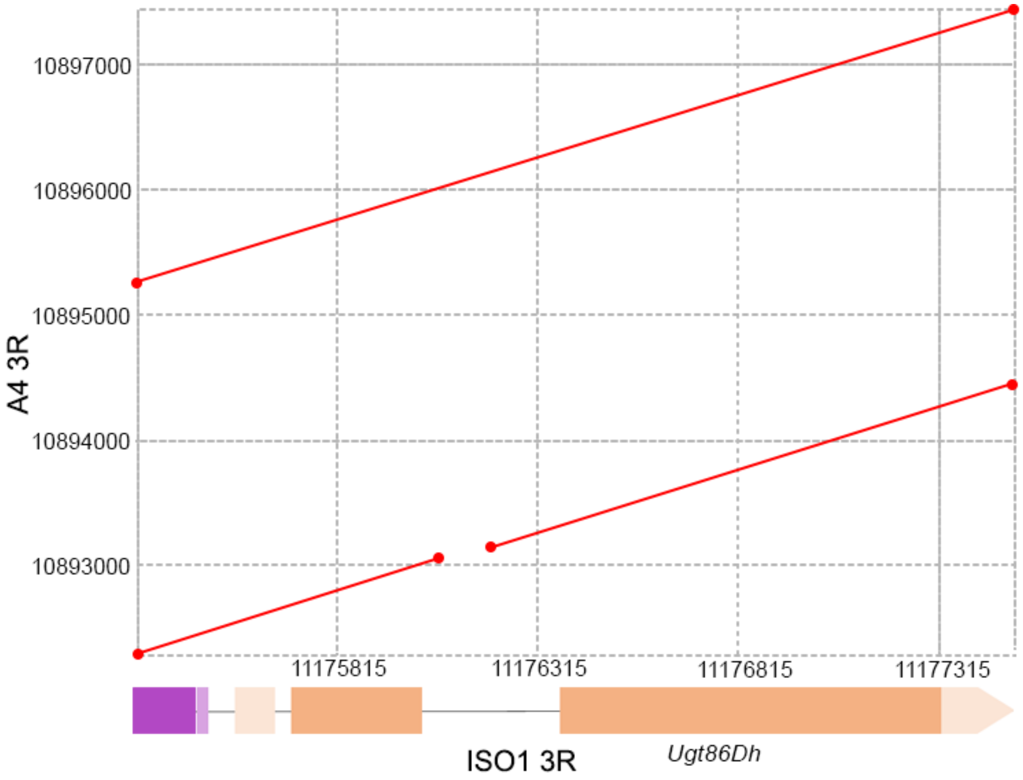

Supplementary Figure 42. Dot plot between ISO1 genomic region 3R:11,175,315-11,177,501 and A4 genomic region 3R: 10,892,303-10,897,452 showing duplication of *Ugt86Dh* in A4. One of the copies in A4 has a deletion within the second intron.

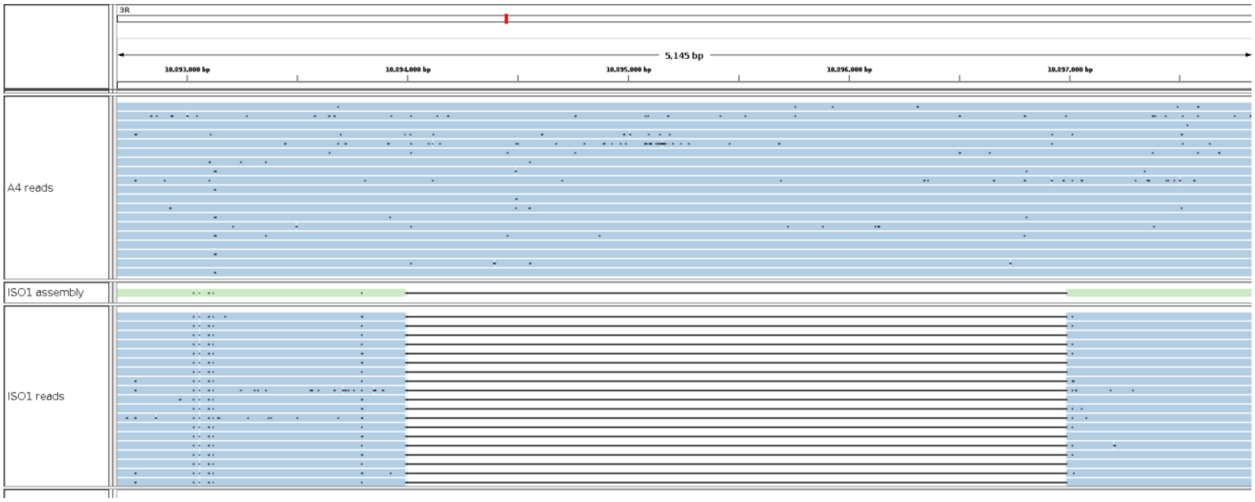

Supplementary Figure 43. Alignment of A4 long reads, ISO1 assembly, and ISO1 long reads to the ISO1 genomic region containing the two copies of *Ugt86Dh*. The alignment gap in ISO1 assembly and ISO1 long reads is due to the absence of the second copy of *Ugt86Dh* in ISO1.

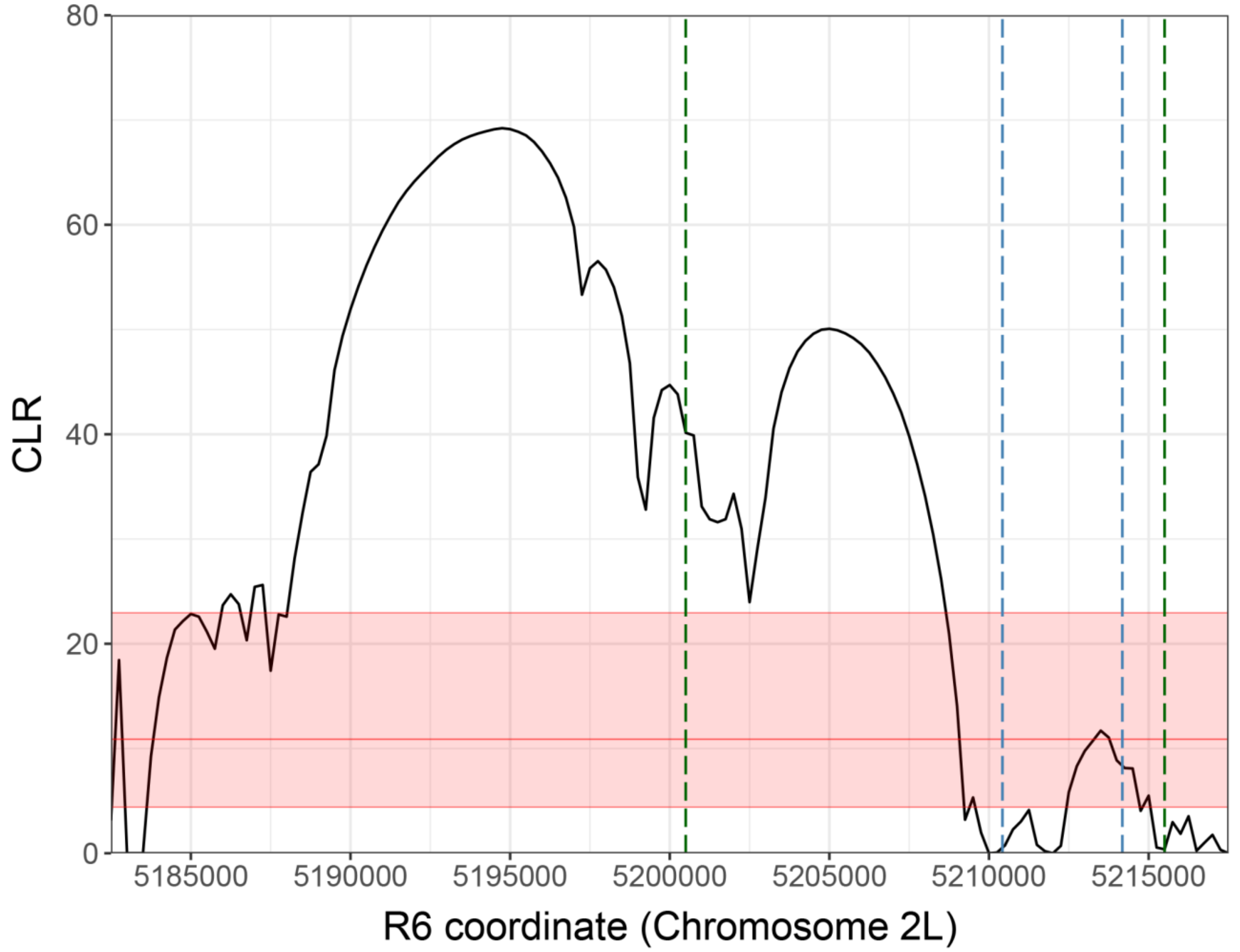

Supplementary Figure 44. Distribution of composite likelihood ratio statistic[14] of the SNP site frequency spectrum at the genomic region containing *Cyp28d1* in the French population from [15] (n_{max} = 20). The CLR peak falls immediately adjacent to the duplication. Given problems genotyping SNPs in duplicates, we do not actually expect the paralogous regions to be easily interpretable in a CLR framework. The red shaded region represents the empirical 95% confidence interval for the maximum CLR values based on 100 neutral simulations using the observed SNPs at this region (Supplementary Methods). Vertical green lines indicate the span of all duplication alleles observed in all populations surveyed in Fig. 3a. Vertical blue lines indicate the extent of the duplication discovered in A4.

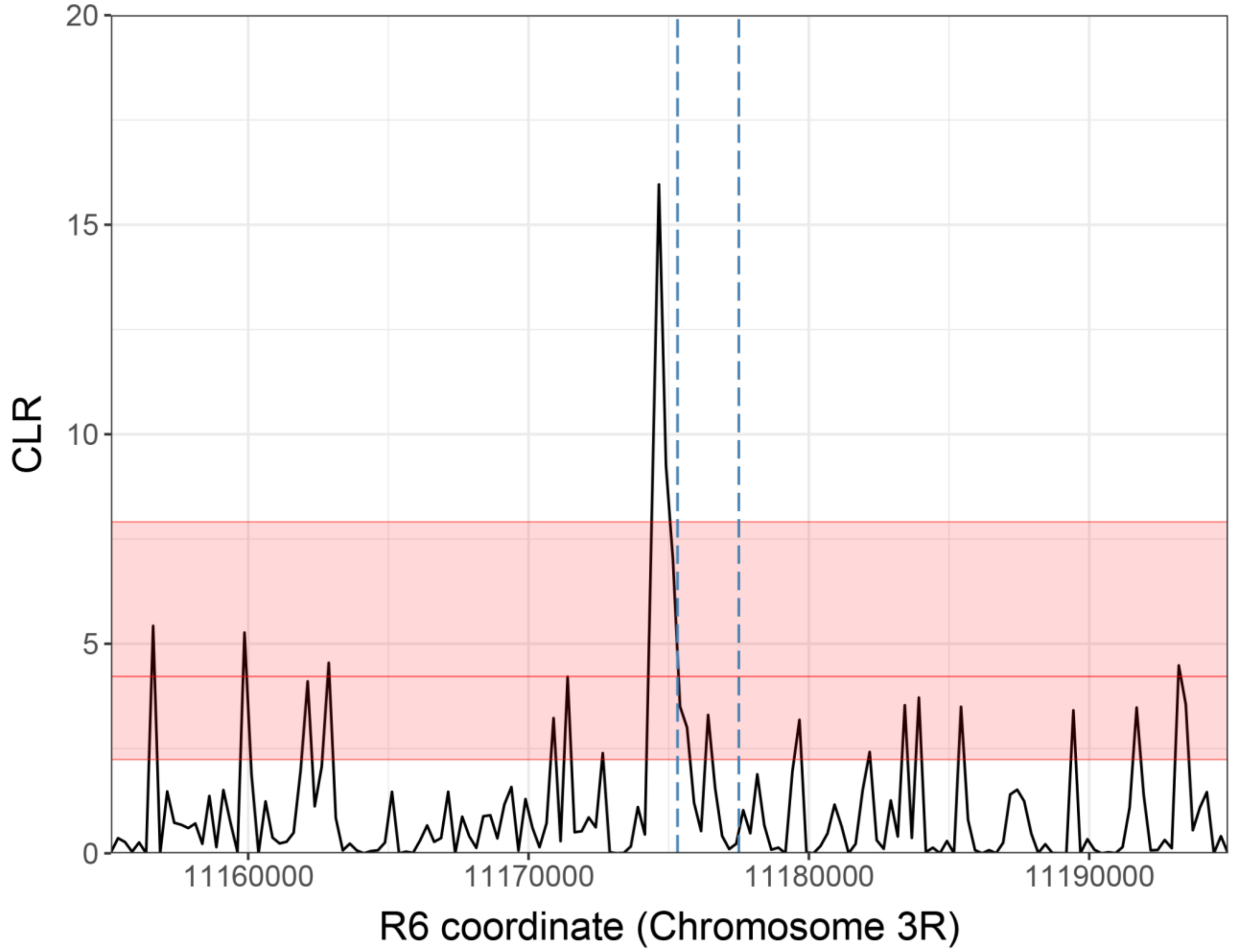

Supplementary Figure 45. Distribution of the CLR statistic[57] at the genomic region containing the *Ugt86Dh* gene duplication in the sub-Saharan African populations, using SNPs called from [16] (n_{max} = 340). The CLR peak falls immediately adjacent to the duplication. Given problems genotyping SNPs in duplicates, we do not actually expect the paralogous regions to be easily interpretable in a CLR framework. The shaded region represents the empirical 95% confidence interval for the maximum CLR values based on 100 neutral simulations using the SNPs present in this genomic region. Vertical blue lines indicate the breakpoints of the observed duplication.

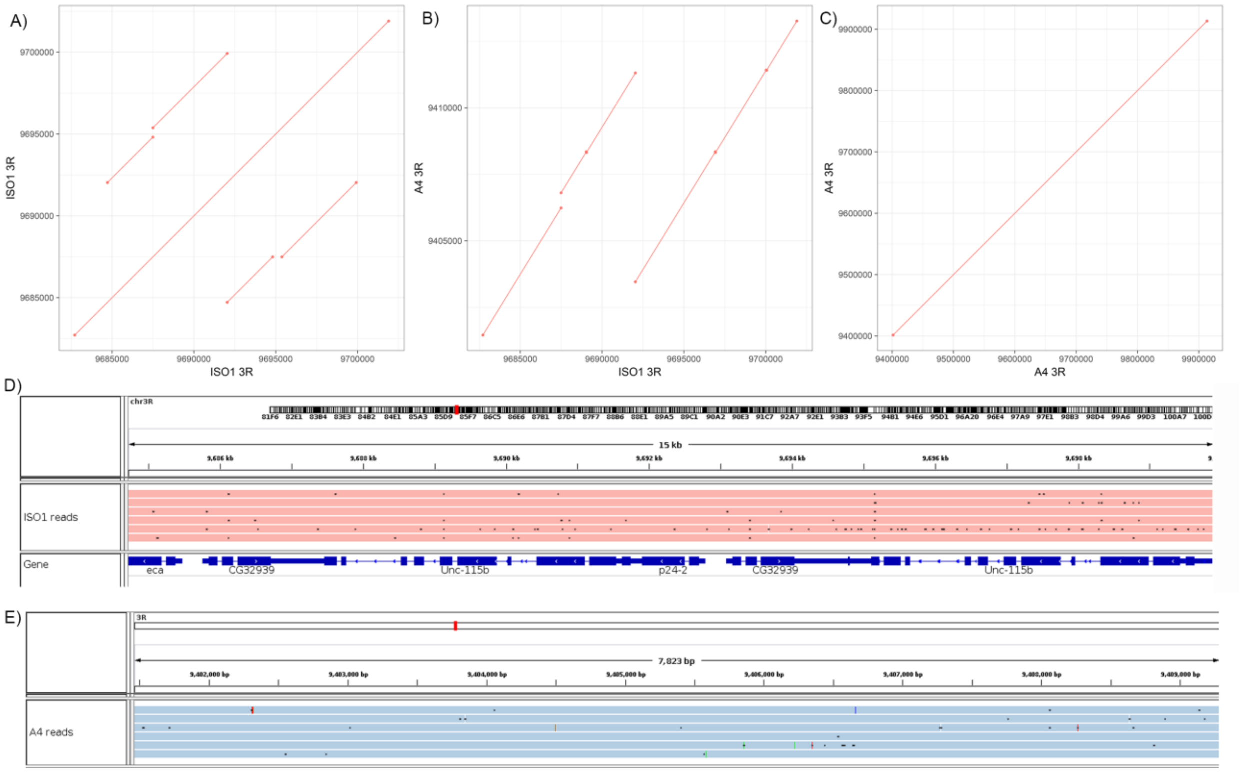

Supplementary Figure 46. Absence of *p24-2* in A4. A) Alignment dot plot between the ISO1 genomic region containing the entire tandem duplication that includes *eca* and *p24-2*. The off-diagonal lines show the location and span of the individual copies. **B)** Alignment dot plot between ISO1 genomic region shown in A and corresponding genomic region from A4. The large break in the diagonal indicate extra sequence in the ISO1 genome and the overlapping diagonals on the two sides of the breaks show that the extra sequence is due to a duplication in ISO1. The overlapping segment of the parallel diagonals is the sequence in A4 that is duplicated in ISO1. The single copy sequence in A4 resembles the distal copy (containing *eca*) in ISO1 more than the proximal copy (containing *p24-2*). **C)** Alignment dot plot of the A4 genomic region as shown in B with itself. Absence of any off-diagonal line shows that A4 does not have the duplicate. **D)** ISO1 PacBio reads aligned to the genomic region containing the tandem duplicates shown in A and B. **E)** A4 PacBio reads aligned to the A4 genomic region containing the single copy sequence shown in C. These spanning reads support the assembly in those regions.

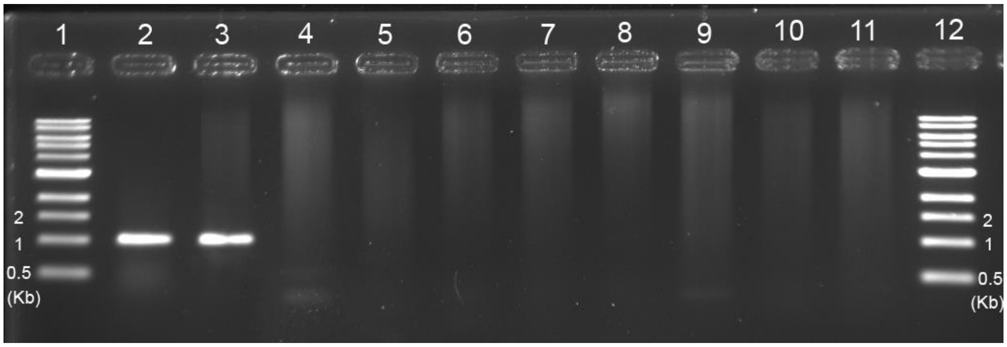

Supplementary Figure 47. Gel image showing presence and absence of *p24-2* in ISO1, Oregon-R, and 8 DSPR strains. From left to right: 1 and 12: 1 Kb ladder from New England Biolabs. 2: PCR with ISO1 DNA and primers spanning the duplication breakpoint in ISO1 produces a 1 kbp amplicon. The remaining lanes show results from PCR with the same primers in different strains: 3: A2; 4: A3; 5: A4; 6: A7; 7:B1; 8: B2; 9:B3; 10: B4; 11: Oregon-R. Evidently, A4, like most other DSPR strains, does not have the duplicate and therefore lacks *p24-2*.

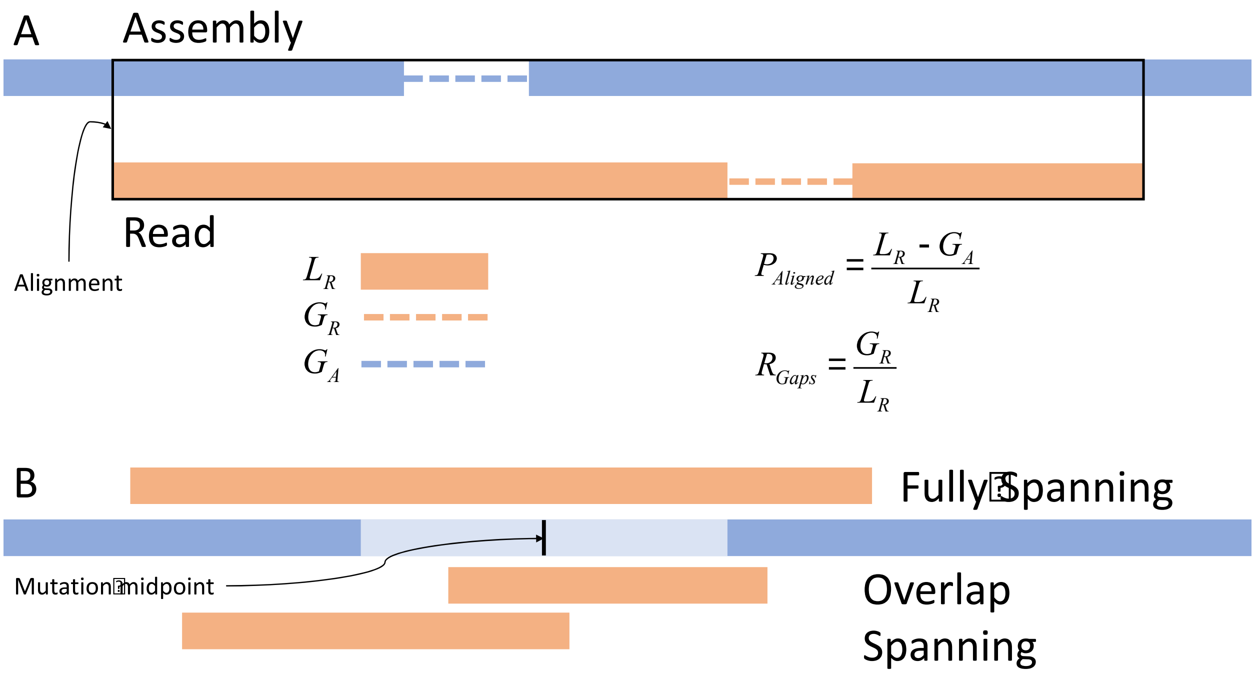

Supplementary Figure 48. Calculation of read alignment metrics used for validations of the SVs. A) Solid bars indicate nucleotide sequence. Dotted lines indicate gaps inserted during alignment. $P_{Aligned}$ is the proportion of nucleotides in the read aligned to nucleotides in the assembly. R_{Gaps} is the ratio of gaps inserted into the read (G_R) over the total length of the read (L_R). **B)** Illustration of reads the fully span a mutation and pairs of overlapping reads that span a mutation together. The mutation is indicated in light blue. The black vertical line is the midpoint of the mutation, which needs to be spanned from both sides to identify overlap spanning pairs. **A-B)** Warm colors are associated with reads. Cool colors are associated with genome assemblies.

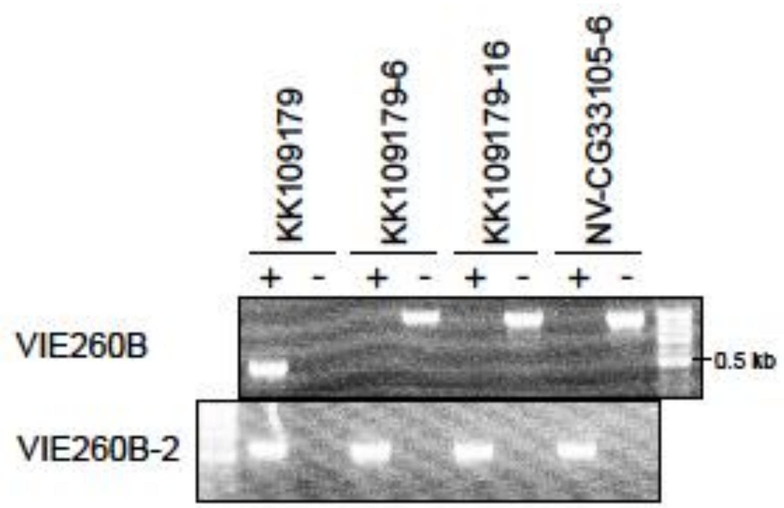

Supplementary Figure 49. Confirmation of RNAi construct presence or absence at VIE260B (2L:22,019,296) and VIE260B-2 (2L:9,437,482) in the original and corrected KK109179 lines and a new line. Presence and absence of the construct was confirmed following an existing protocol for screening for the insertion (see Online Methods for more details). While KK109179 contained insertions at both sites, the corrected and new lines only contain an insertion at VIE260B-2. "+" indicates construct presence; "-" indicates construct absence.

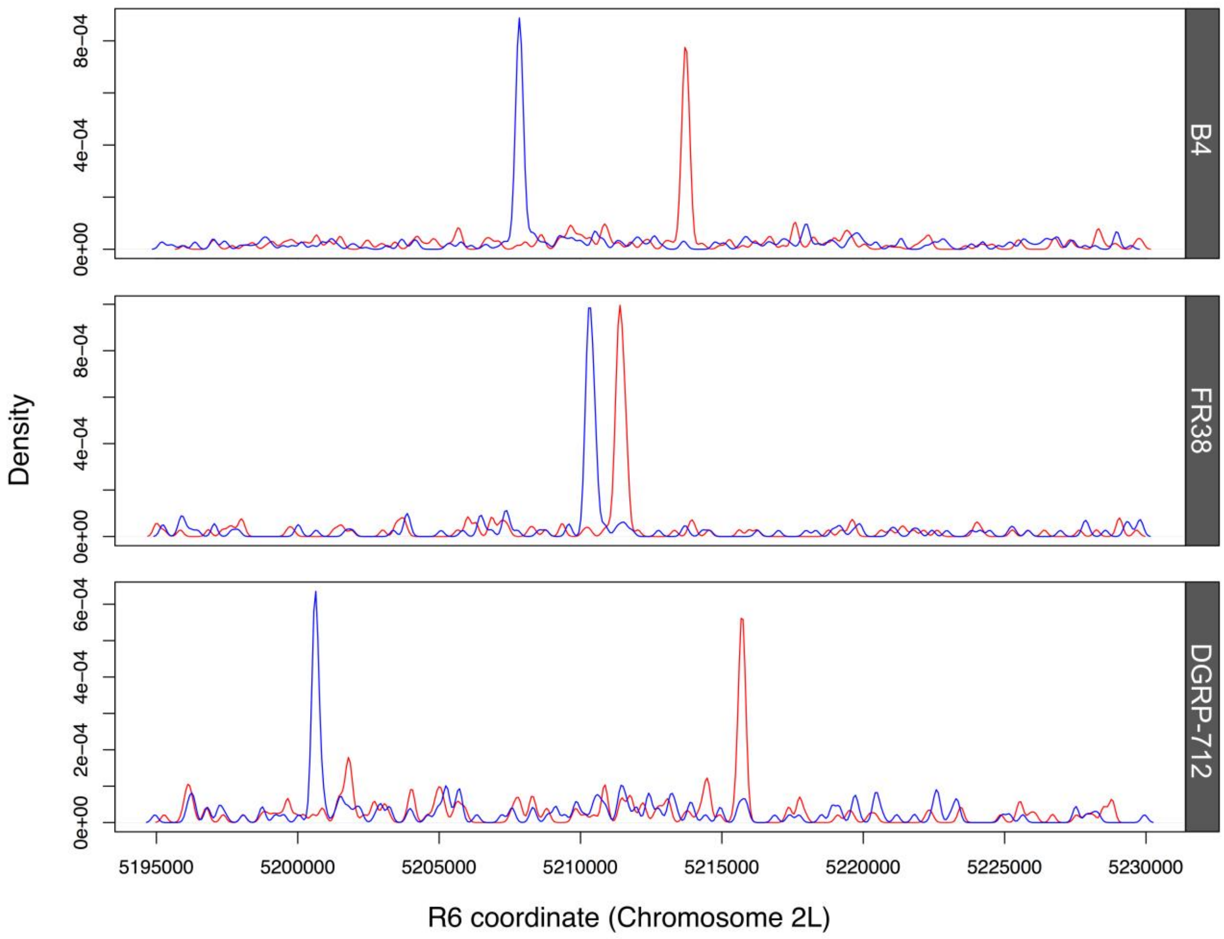

Supplementary Figure 50. Divergent read pair coverage for several alleles at the *Cyp28d1* locus. Samples (top to bottom) are from Zimbabwe (DSPR), Riverside (DSPR), France[15], and North Carolina[17]. Red indicates reads aligning to the forward strand and blue represents the reads aligning to the reverse strand. Unlike the allele in A4, these mutations are not hidden.

Supplementary Table 1. Comparison of assembly metrics between the A4 assembly and the release 6 of the reference assembly (ISO1). The mitochondrial genome was excluded from both genomes and the Y-chromosome sequences were excluded from the ISO1 genome (the A4 genome has no Y-chromosome sequence, as it was assembled from reads derived from females).

Metric	ISO1	A4
**Assembly size (bp)**	139,543,958	144,107,024
**Contig N50 (bp)**	21,485,538	22,302,559
**Scaffold N50 (bp)**	25,286,936	25,479,258
**Number contigs**	2,177	193
**Number scaffolds**	1,856	159
**Complete BUSCO**	2,625	2,621
Single Copy	2,492	2,495
Duplicated	133	126
**Fragmented BUSCO**	29	33
**Missing BUSCO**	21	21

Supplementary Table 2. (Table S2.xlsx) Validation summary of the genomic intervals containing SVs. Genomic intervals possessing multiple SVs of same category were merged prior to the calculation of validation metrics (Supplementary Fig. 48) based on read alignments to those intervals.

Supplementary Table 3. Chromosomal segments used for analyzing functional significance of the structural mutations (heterochromatic sequences are excluded following the coordinates in [18].

Chromosome	Start	End
2L	1	22200000
2R	4700000	25479258
3L	1	23400000
3R	3000000	31815305
X	1	21900000

Supplementary Table 4. Coordinates of the CNVs, indels, and inversions in A4 and ISO1. (Table S4.xlsx)

Supplementary Table 5. CNVs and indels for chromosome arms 2L as called by various CNV (*Pindel*[11], *Pecnv*[12], and TE insertion[5] calling software. (Table S5.xlsx)

Supplementary Table 6. Summary of putative TE introns. (Table S6.xlsx)

Supplementary Table 7. Genes mutated by non-TE indels in A4 (Table S7.xlsx).

Supplementary Table 8. Expression changes in genes in A4 with increased copy number. (Table S8.xlsx)

Supplementary Table 9. A4 duplicated genes with putative adaptive role.

Gene Symbol	FlyBase ID	Expression change (Log_2)	Phenotype	Detected by Illumina reads
CG31157	FBgn0051157	2.53	Cold resistance[19]	Yes
CG4302	FBgn0027073	2.18	Cold adaptation[20] Caffeine resistance[21]	Yes
CG6912	FBgn0038290	1.06	Feeding preference[22]	Yes
CG7966	FBgn0038115	1.72	Cold resistance[20,23]	Yes
Cyp28d1	FBgn0031689	5.74	Nicotine resistance[7]	No
Or85f	FBgn0037685	3.42	Olfaction[24]	Yes
Qtzl	FBgn0051864	0.52	Fertility/nervous system[25]	No
Ugt86Dh	FBgn0040252	1.29	Nicotine resistance[7] DDT resistance[26]	No

Supplementary Table 10. Number of offspring produced by flies with constitutive *p24-2* RNAi. KK109179-6 and KK109179-16 lines are produced after crossing out the ectopic RNAi integration site in *tio* from the VDRC line KK109179 (Supplementary Fig. 49). NV-CG33105-2 and NV-CG33105-6 are new *p24-2* RNAi lines that were created in our study. 601000_CTRL is the VDRC strain which was used as the parental line for all VDRC KK RNAi lines. For statistical tests, the control wild type to balancer offspring ratio for beta-tubulin and actin drivers used were 53:47 and 55:45, respectively.

	Tub::GAL				Act::GAL			
Line	Balancer	RNAi (wt)	Chi-square	*p,* 1 d.f.	Balancer	RNAi (wt)	Chi-square	*p*, 1 d.f.
KK109179-6	65	60	0.073	0.79	86	80	2.964	0.09
KK109179-16	70	74	1.221	0.27	91	102	0.310	0.58
NV-CG33105-2	102	106	1.451	0.23	115	126	0.638	0.42
NV-CG33105-6	77	82	1.458	0.23	134	140	1.554	0.21
60100_CTRL	664	755	-	-	1021	1240	-	-

Supplementary Table 11. The unmerged CNV calls from A4-ISO1 genome alignment.

Supplementary Table 12. PCR primers for *p24-2* RNAi line.

**Primer Name**	**Sequence (3’→5’)**
Cyp28d1_prox_F	CAGAGTTTTACGGATAATCCG
Cyp28d1_prox_R	ACCGATCTCCTCCCTCAACT
Cyp28d1_dist_F	GCAGAAGTGCATCCAAGTT
Cyp28d1_dist_R	ACGCTCACTCCGTTTTTGTT
p24-2_pres_R	CCAACCTTGCCCCGTTTTAC
p24-2_pres_R	CATCAGCCTGGCCCTGATTC
CG33105_RNAi_F	AACGAGCAGTGTTTGTGTGTG
CG33105_RNAi_R	ATCATTGATAAGGCCAAGGG
CG33105-pres-F	CATGCATGTGGAAGTACGCG
CG33105-pres-R	CGTTCCCAATGTGCTAGGGT

Supplementary note:

Alignment between sequences of *eca*, *p24-2*, and the hairpin of two VDRC RNAi constructs for *eca* (GD17660) and *p24-2* (GD2877). The latter (GD2877) was used by Chen et al. 2010 to knock down *p24-2*. As evidenced in the alignment, GD2877 hairpin sequence is 100% identical to both *eca* and *p24-2* and therefore it is not specific to *p24-2*.

```
eca-RA/1-946         369 TCTCGTTCACCTCGCACACTCCTGGCGAGCACGTCATCTGCATGTTCTCGAACAGC 424
p24-2-RA/1-830       367 TCTCGTTCACCTCGCACACTCCTGGCGAGCACGTCATCTGCATGTTCTCGAACAGC 422
GD2877_p24-2/1-333     1 TCTCGTTCACCTCGCACACTCCTGGCGAGCACGTCATCTGCATGTTCTCGAACAGC 56
GD17660_eca/1-333      1 TCTCGTTCACCTCGCACACTCCTGGCGAGCACGTCATCTGCATGTTCTCGAACAGC 56

eca-RA/1-946         425 ACCGCGTGGTTCAGTGGTGCCCAGCTGCGTGTTCACCTGGACATCCAGGTGGGAGA 480
p24-2-RA/1-830       423 ACCGCGTGGTTCAGTGGTGCCCAGCTGCGTGTTCACCTGGACATCCAGGTGGGAGA 478
GD2877_p24-2/1-333    57 ACCGCGTGGTTCAGTGGTGCCCAGCTGCGTGTTCACCTGGACATCCAGGTGGGAGA 112
GD17660_eca/1-333     57 ACCGCGTGGTTCAGTGGTGCCCAGCTGCGTGTTCACCTGGACATCCAGGTGGGAGA 112

eca-RA/1-946         481 GCACGCTATCGACTACGCCCATGTGGCGCAGAAGGAGAAACTGACTGAGCTGCAGC 536
p24-2-RA/1-830       479 GCACGCTATCGACTACGCCCATGTGGCGCAGAAGGAGAAACTGACTGAGCTGCAGC 534
GD2877_p24-2/1-333   113 GCACGCTATCGACTACGCCCATGTGGCGCAGAAGGAGAAACTGACTGAGCTGCAGC 168
GD17660_eca/1-333    113 GCACGCTATCGACTACGCCCATGTGGCGCAGAAGGAGAAACTGACTGAGCTGCAGC 168

eca-RA/1-946         537 TGCGCATCCGCCAGCTACTTGACCAGGTGGAGCAGATCACCAAGGAGCAGAACTAC 592
p24-2-RA/1-830       535 TGCGCATCCGCCAGCTACTTGACCAGGTGGAGCAGATCACCAAGGAGCAGAACTAC 590
GD2877_p24-2/1-333   169 TGCGCATCCGCCAGCTACTTGACCAGGTGGAGCAGATCACCAAGGAGCAGAACTAC 224
GD17660_eca/1-333    169 TGCGCATCCGCCAGCTACTTGACCAGGTGGAGCAGATCACCAAGGAGCAGAACTAC 224

eca-RA/1-946         593 CAGCGATACCGCGAGGAGCGGTTCCGTCACACCAGCGAGAGCACCAACTCCCGCGT 648
p24-2-RA/1-830       591 CAGCGATACCGCGAGGAGCGGTTCCGTCACACCAGCGAGAGCACCAACTCCCGCGT 646
GD2877_p24-2/1-333   225 CAGCGATACCGCGAGGAGCGGTTCCGTCACACCAGCGAGAGCACCAACTCCCGCGT 280
GD17660_eca/1-333    225 CAGCGATACCGCGAGGAGCGGTTCCGTCACACCAGCGAGAGCACCAACTCCCGCGT 280

eca-RA/1-946         649 GCTCTGGTGGTCGCTGGCCCAGACCGTCGTCTTGGTTTGCATGGGCTTCTGGC    946
p24-2-RA/1-830       647 GCTCTGGTGGTCGCTGGCCCAGACCGTCGTCTTGGTTTGCATGGGCTTCTGGC    830
GD2877_p24-2/1-333   281 GCTCTGGTGGTCGCTGGCCCAGACCGTCGTCTTGGTTTGCATGGGCTTCTGGC    333
GD17660_eca/1-333    281 GCTCTGGTGGTCGCTGGCCCAGACCGTCGTCTTGGTTTGCATGGGCTTCTGGC    333
```

Illumina data

Illumina reads used to determine *Cyp6a17* deletion, *Cyp28d1* and *Ugt86Dh* duplicates frequencies were obtained from the following sources: DPGP2[27] (Cameroon, Ethiopia, Kenya, Rwanda, Gabon, Guinea, South Africa), DPGP3[15] (Zambia), France and Georgia[16], GDL[28] (Netherlands, New York), and DGRP[17] (North Carolina).

Supplementary References

1. Hoskins, R.A. *et al.* The Release 6 reference sequence of the Drosophila melanogaster genome. *Genome research* **25**, 445-58 (2015).
2. Kim, K.E. *et al.* Long-read, whole-genome shotgun sequence data for five model organisms. *Sci Data* **1**, 140045 (2014).
3. dos Santos, G. *et al.* FlyBase: introduction of the Drosophila melanogaster Release 6 reference genome assembly and large-scale migration of genome annotations. *Nucleic Acids Res* **43**, D690-7 (2015).
4. Nitasaka, E., Yamazaki, T. & Green, M.M. The Molecular Analysis of Brown Eye Color Mutations Isolated from Geographically Discrete Populations of Drosophila-Melanogaster. *Molecular & General Genetics* **247**, 164-168 (1995).
5. Cridland, J.M. & Thornton, K.R. Validation of rearrangement break points identified by paired-end sequencing in natural populations of Drosophila melanogaster. *Genome Biol Evol* **2**, 83-101 (2010).
6. Cridland, J.M., Macdonald, S.J., Long, A.D. & Thornton, K.R. Abundance and distribution of transposable elements in two Drosophila QTL mapping resources. *Mol Biol Evol* **30**, 2311-27 (2013).
7. Marriage, T.N., King, E.G., Long, A.D. & Macdonald, S.J. Fine-mapping nicotine resistance loci in Drosophila using a multiparent advanced generation inter-cross population. *Genetics* **198**, 45-57 (2014).
8. Swinburne, I.A. & Silver, P.A. Intron delays and transcriptional timing during development. *Dev Cell* **14**, 324-30 (2008).
9. Stapleton, M. *et al.* The Drosophila gene collection: identification of putative full-length cDNAs for 70% of D. melanogaster genes. *Genome Res* **12**, 1294-300 (2002).
10. Najarro, M.A. *et al.* Identifying Loci Contributing to Natural Variation in Xenobiotic Resistance in Drosophila. *PLoS Genet* **11**, e1005663 (2015).
11. Ye, K., Schulz, M.H., Long, Q., Apweiler, R. & Ning, Z. Pindel: a pattern growth approach to detect break points of large deletions and medium sized insertions from paired-end short reads. *Bioinformatics* **25**, 2865-71 (2009).
12. Rogers, R.L. *et al.* Landscape of standing variation for tandem duplications in Drosophila yakuba and Drosophila simulans. *Mol Biol Evol* **31**, 1750-66 (2014).
13. Abyzov, A., Urban, A.E., Snyder, M. & Gerstein, M. CNVnator: An approach to discover, genotype, and characterize typical and atypical CNVs from family and population genome sequencing. *Genome Research* **21**, 974-984 (2011).
14. Nielsen, R. *et al.* Genomic scans for selective sweeps using SNP data. *Genome Res* **15**, 1566-75 (2005).
15. Bergman, C.M. & Haddrill, P.R. Strain-specific and pooled genome sequences for populations of Drosophila melanogaster from three continents. *F1000Res* **4**, 31 (2015).
16. Lack, J.B. *et al.* The Drosophila genome nexus: a population genomic resource of 623 Drosophila melanogaster genomes, including 197 from a single ancestral range population. *Genetics* **199**, 1229-41 (2015).
17. Mackay, T.F.C. *et al.* The Drosophila melanogaster Genetic Reference Panel. *Nature* **482**, 173-178 (2012).

18. Hoskins, R.A. *et al.* Heterochromatic sequences in a Drosophila whole-genome shotgun assembly. *Genome Biol* **3**, RESEARCH0085 (2002).
19. Huylmans, A.K. & Parsch, J. Population- and sex-biased gene expression in the excretion organs of Drosophila melanogaster. *G3 (Bethesda)* **4**, 2307-15 (2014).
20. Telonis-Scott, M., Hallas, R., McKechnie, S.W., Wee, C.W. & Hoffmann, A.A. Selection for cold resistance alters gene transcript levels in Drosophila melanogaster. *Journal of Insect Physiology* **55**, 549-555 (2009).
21. Coelho, A. *et al.* Cytochrome P450-Dependent Metabolism of Caffeine in Drosophila melanogaster. *Plos One* **10**(2015).
22. Toshima, N., Hara, C., Scholz, C.J. & Tanimura, T. Genetic variation in food choice behaviour of amino acid-deprived Drosophila. *Journal of Insect Physiology* **69**, 89-94 (2014).
23. Turner, T.L., Levine, M.T., Eckert, M.L. & Begun, D.J. Genomic analysis of adaptive differentiation in Drosophila melanogaster. *Genetics* **179**, 455-473 (2008).
24. Rollmann, S.M. *et al.* Odorant Receptor Polymorphisms and Natural Variation in Olfactory Behavior in Drosophila melanogaster. *Genetics* **186**, 687-U364 (2010).
25. Rogers, R.L., Bedford, T., Lyons, A.M. & Hartl, D.L. Adaptive impact of the chimeric gene Quetzalcoatl in Drosophila melanogaster. *Proceedings of the National Academy of Sciences of the United States of America* **107**, 10943-10948 (2010).
26. Pedra, J.H.F., McIntyre, L.M., Scharf, M.E. & Pittendrigh, B.R. Genom-wide transcription profile of field- and laboratory-selected dichlorodiphenyltrichloroethane (DDT)-resistant Drosophila. *Proceedings of the National Academy of Sciences of the United States of America* **101**, 7034-7039 (2004).
27. Pool, J.E. *et al.* Population Genomics of sub-saharan Drosophila melanogaster: African diversity and non-African admixture. *PLoS Genet* **8**, e1003080 (2012).
28. Grenier, J.K. *et al.* Global diversity lines - a five-continent reference panel of sequenced Drosophila melanogaster strains. *G3 (Bethesda)* **5**, 593-603 (2015).
